# Both tumour cells and infiltrating T-cells in equine sarcoids express FOXP3 associated with an immune-supressed cytokine microenvironment

**DOI:** 10.1186/s13567-016-0339-8

**Published:** 2016-05-09

**Authors:** A. Douglas Wilson, Chelsea Hicks

**Affiliations:** School of Veterinary Sciences, University of Bristol, Langford, Bristol, BS40 5DU UK

## Abstract

Bovine papillomavirus (BPV) infections of equine species have a central role in the aetiology of equine sarcoids; a common benign skin tumour of horses, zebras and donkeys. Within the lesions, all of the early papillomavirus genes are expressed and promote the excessive replication of fibroblasts which characterise these tumours. Equine sarcoids differ from BPV induced fibro-papillomas of cattle (the natural host of BPV), in that they do not produce high amounts of virus particles, do not usually regress spontaneously and do not sero-convert to BPV; features which suggest that affected horses lack an effective anti-viral immune response to BPV. Equine sarcoids contain large numbers of CD4+ CD8+ dual positive T-cells which uniformly express FOXP3, the key transcription factor of regulatory T-cells, and FOXP3 is also expressed within the BPV infected fibroblasts. Compared to healthy skin, sarcoids showed increased mRNA transcription for FOXP3 and the regulatory cytokine TGFβ. Transcription of IL17, which has been shown to have a regulatory function in human papillomavirus-associated tumours, was also elevated in equine sarcoids compared to spleen. In contrast, the levels of mRNA transcripts for effector T cell cytokines IL2, IL4 and interferon-gamma (IFNγ) were not elevated in sarcoids compared to healthy skin or spleen. Similarly neither interferon-alpha (IFNα), interferon-beta (IFNβ) nor IL12 family members were elevated in sarcoids compared to normal skin. We suggest that the regulatory cytokine micro-environment within sarcoids enables the persistence of the lesions by preventing an effective anti-viral immune response.

**Electronic supplementary material:**

The online version of this article (doi:10.1186/s13567-016-0339-8) contains supplementary material, which is available to authorized users.

## Introduction

Papillomaviruses are among the most widespread of animal viruses, with many hosts harbouring multiple virus types. Phylogenetic studies show that the papillomaviruses within a specific host often have a degree of similarity, indicating that host and virus have co-evolved over a prolonged period of time during which new variants slowly emerged as mutations accumulated within the virus genome [1]. Accordingly, it is assumed that the majority of papillomaviruses are host-specific and do not readily cross species barriers. However, phylogenetic studies have also revealed cases where closely related papillomavirus are found in separate host species indicating that transfer of papillomavirus infections across species does occur and may lead to the [more rapid] emergence of new papillomavirus types [1]. Bovine papillomavirus 1 and 2 (BPV1, BPV2) provide examples of cross species infection, in that both virus types cause fibropapilloma lesions in cattle (*Bos taurus*) and are also associated with the occurrence of sarcoids in domestic horses (*Equus caballus*), donkeys (*Equus asinus*) and zebras (*Equus zebra*) [2–4]. In equine spp. sarcoids is a collective term for a group of non-resolving pathological skin lesions, characterised by proliferation of fibroblasts along with the presence of DNA from BPV1 or BPV2: together these lesions comprise the commonest skin tumours in equines [5].

The exact nature of the link between BPV and sarcoids has proved controversial as electron microscopy studies and immunohistology of formalin fixed tissues which detect virus particles and late virus proteins in bovine fibropapillomas; have consistently failed to demonstrate the presence of BPV capsids or late proteins within sarcoids [6–9].

Early attempts to transmit BPV by directly inoculating cell free papilloma material derived from cattle into horses did indeed induce lesions characterised by fibroblast proliferation which morphologically resembled sarcoids. However, BPV could not generally be passaged from these sarcoid type lesions into new horses [10, 11] indicating an absence of continuing infectious virus particle production. In contrast, lesions of cattle infected with either BPV1 or BPV2 contain large numbers of virus particles [8], which when isolated can readily be transmitted to new hosts in vivo [12]. Similarly, transmission studies using material taken from equine sarcoids found that while explants of sarcoid tissue could sometimes be transmitted within and between equine hosts, injection of cell free supernatants or minced sarcoid material between horses did not reliably transmit sarcoids and was only occasionally successful when the material was introduced to scarified skin [13]. Overall the experiments could not clearly distinguish between the de-novo transfer of infectious virus between equine hosts and the reactivation of established latent papillomavirus infection subsequent to scarification such as has been described in cattle [3].

The lesions produced by experimental inoculation of horses with BPV particles derived from cattle regressed spontaneously after several weeks [10, 11] in the same manner as naturally occurring BPV fibropapillomas in cattle, which resolve spontaneously after several months in association with the appearance of tumour infiltrating leukocytes [3, 14]. In contrast, naturally occurring sarcoids in horses typically persist indefinitely and frequently re-grow following surgical removal [15]. Early experiments showed that regression of experimental sarcoids induced following inoculation of horses with BPV derived from cattle was accompanied by the appearance of antibodies to BPV, yet no anti-BPV antibodies were seen in naturally occurring sarcoids, observations which at the time caused the authors to cast doubt on the role of BPV in the aetiology of equine sarcoids [16, 17]. Nevertheless, sarcoids invariably contain BPV1 or BPV2 DNA, present as an un-integrated episome [18] and several early transforming papillomavirus proteins are expressed in sarcoid tissues, supporting the role of BPV as the aetiological agent responsible for sarcoids [9, 19–21]. In addition, mRNA transcripts encoding both early and late virus proteins have been detected by RT-PCR of equine sarcoid tissues [9, 22], although neither study showed the late gene mRNA to be in the form of a correctly spliced transcript that could be translated into a functional protein. In cattle, late protein synthesis and productive virus infection takes place at the junction between the upper stratum spinosum and keratinised layers of the epidermis [9, 23]. Two studies have reported the detection of late BPV capsid antigen expression in a minority of equine sarcoids stained by immunofluorescence using frozen sections [9, 19], but as neither study showed the clear nuclear staining that is seen in other productive papillomavirus infections the results remain inconclusive. Localisation of BPV DNA by in situ hybridisation detected virus exclusively in the fibroblasts and not in the overlying epidermis [24], in contradiction later PCR studies showed BPV DNA to be present throughout the epidermal layers of equine sarcoids [19, 25]. However a recent study using a highly sensitive amplified in situ hybridisation technique also failed to detect viral nucleic acid within the nuclei of epidermal cells overlying sarcoid lesions in the majority of cases, while once again BPV nucleic acid was invariably present in the fibroblast layers [26]. Interestingly, in just a few cases BPV positive nuclei were seen in scattered epidermal cells as well as in sebocytes and apocrine sweat glands [26], locations not previously associated with papillomavirus infection. Small amounts of BPV DNA combined with late virus protein have been detected in a subset of sarcoid cases by immuno-capture PCR using antibodies specific for BPV capsid antigens [27] providing possible evidence for trace amounts of DNA containing virus particles within a subset of equine sarcoids.

Despite the lack of conclusive evidence for efficient production of infectious BPV virions in sarcoids, analysis of the genomes of BPV1 present in sarcoids has shown distinct equine associated sequence variations within the E5, E2 genes and long control regions [28, 29], moreover BPV1-DNA isolated from horses consistently contained a deletion of four residues from within the L2 protein that may also indicate a distinctive equine sub-type of BPV1 has evolved [9]. The evolution and maintenance of such equine specific variants would require sequential passage of BPV through horses, yet to date the evidence for productive virus evidence remains scant. One explanation that could reconcile the conflicting lines of evidence is that the prolonged survival of sarcoids, in which production of virus particles is intermittent or falls below the limit of detection by conventional immune-histology or electron microscopy, would nonetheless permit a minimal transfer of virus between horses (basic reproduction number R0 ≥1) sufficient to sustain an endemic infection. However, persistent infection requires a further conjecture to account for the failure of the immune system of horses to eliminate the virus compared to BPV lesions of cattle [3, 14].

The demonstration that BPV E5 protein can inhibit the surface expression of MHC class I [30], and that BPV early genes also down-regulate TLR4 expression [31] provide mechanisms for immune evasion which go some way to explaining sarcoid persistence, but leaves the problem of explaining why BPV E5 does not act in a similar manner to prevent ultimate regression of papillomas in their natural bovine host. Further evidence for a suppressed immune response contributing to sarcoid persistence in horses has recently been provided by a study in which mRNA encoding the regulatory T-cell transcription factor FOXP3 along with certain other cytokines were shown to be elevated within sarcoid tumours [32].

In this paper prompted by our earlier observation that sarcoid tissue is infiltrated by large numbers of T-cells [9] we further examine the proposition that the cytokine micro-environment and infiltrating regulatory T-cells have a role in blocking the clearance of virus infected cells from sarcoids, thereby facilitating the ultimate transmission of virus between horses and enabling the evolution of equine specific BPV variants.

## Materials and methods

### Sample collection

Formalin-fixed and paraffin wax-embedded samples of sarcoid tissue were retrieved from the pathology archive of the University of Bristol, School of Veterinary Sciences. Fresh sarcoid material was collected from six horses during surgical removal or immediately post mortem; tissues were embedded in optimal cutting temperature (OCT) medium and snap frozen in isopentane cooled over liquid nitrogen, before storage at −70 °C.

Additional material from six fresh sarcoids, was placed in RNAlater^®^ (Invitrogen, UK) stored for 48 h at 4 °C and then kept at −70 °C until used. For comparison six samples of spleen and six samples of healthy skin (from horses without sarcoid lesions) were collected post mortem from an abattoir, placed immediately in RNAlater, then taken to the laboratory for processing.

### Immunohistology

Formalin fixed tissue samples were stained using rabbit anti-CD3, biotinylated donkey anti-rabbit and avidin biotin peroxidase conjugate as previously described [9] FOXP3 expression was detected using either 1:400 affinity purified polyclonal rabbit anti-FOXP3 (ab10563 abCam, UK) which maps to an epitope at the extreme C-terminal of human and equine FOXP3 or monoclonal rat anti mouse Foxp3 (FJK-16 s eBioscience) which maps to an epitope between residues 75-125 in the region of exon2 of murine Foxp3. For both antibodies heat treatment in Tris–EDTA pH 9.0 was used for antigen recovery. For immunofluorescence the primary antibodies used were 3H5 monoclonal mouse anti-BPV E2 (1 μg/mL) [33], and rabbit anti-CD3 (diluted 1:400; A0452 Dako), 1:400 polyclonal rabbit anti-FOXP3 (ab10563 abCam UK), 1 μg/mL monoclonal mouse anti-equine CD4 (HB61A), CD8 (73/6.9.1), MHC I (H58A) and MHC II H34A) (Kingfisher Biotec). Control sections were incubated with normal rabbit immunoglobulins (diluted 1:400; XO936 Dako), or an isotype matched monoclonal antibody in place of the primary antibody. Secondary antibodies were affinity-purified Fab fragments of donkey FITC-anti-mouse or FITC anti-rat and texas red anti-rabbit immunoglobulins absorbed against multiple species serum proteins; (Jackson Immunoresearch) used at 1:1000 dilution. For dual staining with primary mouse monoclonal antibodies, FITC or TRITC conjugated to goat anti-mouse sub-class specific polyclonal antibodies were used at a dilution of 1:100 (Southern-Biotech). For immunofluorescence staining 6 μm thick frozen sections were cut and mounted onto slides coated with chrome alum gelatine, air dried, then fixed in 100% acetone for 5 min at room temperature. Individually wrapped slides were stored at −70 °C prior to use. Sections were equilibrated to room temperature before unwrapping, then re-hydrated in PBS and non-specific protein binding blocked by incubation in PBS with 10% horse serum. Sections were stained with combinations of rabbit and mouse primary antibodies diluted in PBS with 10% horse serum, washed in PBS, followed by the appropriate florescent conjugate antibodies. Slides were mounted in Vectashield^®^ DAPI aqueous mounting medium (Vector Laboratories). Images were taken using a Leica DC350RX camera and Leica Qfluoro software (Leica microsystems UK Ltd. Miltom Keynes, UK). For each antibody combination, control images, in which the primary antibody is replaced by 1:400 rabbit immunoglobulin or 1 μg/mL isotype matched mouse and rat monoclonal antibodies were taken using the same camera settings and showed no specific staining (see Figure 3D for representative image).

### Quantitative reverse transcriptase polymerase chain reaction (RT-QPCR)

Total RNA was isolated from 25 μg tissue stored in RNAlater using a Nuclelospin^®^ RNA II kit (Machery-Nagel) following the manufacturer’s instructions including an on-column DNAse step. To ensure the complete removal of DNA two extra DNAse and re-isolation steps were included as previously described [34]. RNA was quantified using a micro-fluorimiter (Qubit Invitrogen). Fifty nano-grams of total RNA from each sample was reverse transcribed using ImProm-II™ Reverse Transcriptase (Promega) and random hexamer primers according to the manufacturer’s protocol, the resultant cDNA was made up to a final volume of 120 μL in water. Five microlitre aliquots of cDNA were run in PCR reactions consisting of 12.5 μL Gotaq mastermix (Promega), 1.25 μL 50 mM MgCl_2_, 0.5 μL of 10 μM forward and reverse primers plus 0.5 μL of the appropriate 10 μM probe conjugated to 3′FAM and 5′ BQ1 (Metabion) added to each reaction. The primers and probes for multiple housekeeping genes and equine cytokines (Additional file 1) were designed using primer 3 software [35] with a target melting point of 60 °C for primers and 70 °C for probes, optimal 50% CG content, polyX <3 and 3′ complementarity ≤1. The product for each potential primer pair was analysed to ensure the absence of competing secondary structures using m-fold [36]. Following an initial PCR to generate DNA product, the efficiency of each primer was calculated by running a series of ten-fold dilutions of DNA template using syber green in place of the probe on a Stratagene MX3005P thermal cycler. Cycling conditions of 2 min at 95 °C followed by 40 cycles of 10 s at 95 °C and 20 s at 60 °C. The thermal profile for melting curve determination began with an incubation of 1 min at 60 °C followed by a gradual increase in temperature (1 °C/15 s), during which time changes in fluorescence were monitored at 0.5 °C intervals. The resultant melting curve was used to confirm the absence of primer dimers and the MXpro software used to calculate the efficiency of the reactions.

Each sample was run using every cytokine and housekeeping gene primer pair in individual reactions. The data was normalised using GeNorm software [37] to identify the most stable combination of multiple housekeeping genes which in this case were Actβ, HPRT1, RPL32. The normalised data for each individual cytokine are expressed as relative copy number, whereby the sample with the lowest recorded cycle threshold (CT) takes the value 1 and all other samples become a multiple of this reference value. For the purposes of analysis negative samples which did not give a CT (i.e., remained negative after 40 cycles) were set to a dummy variable value of 0.

### Statistical analysis

The cytokine data was analysed using Kruskal–Wallis analysis of variance; where significant differences were detected (*p* ≤ 0.05), we then used a Man-Whitney test with a Bonferroni adjustment of *p* < 0.016 to compare cytokine expression between the pairs of tissues while maintaining an overall significance level of α < 0.05 (Additional file 2).

## Results

### Immunohistology

**Figure 1:**
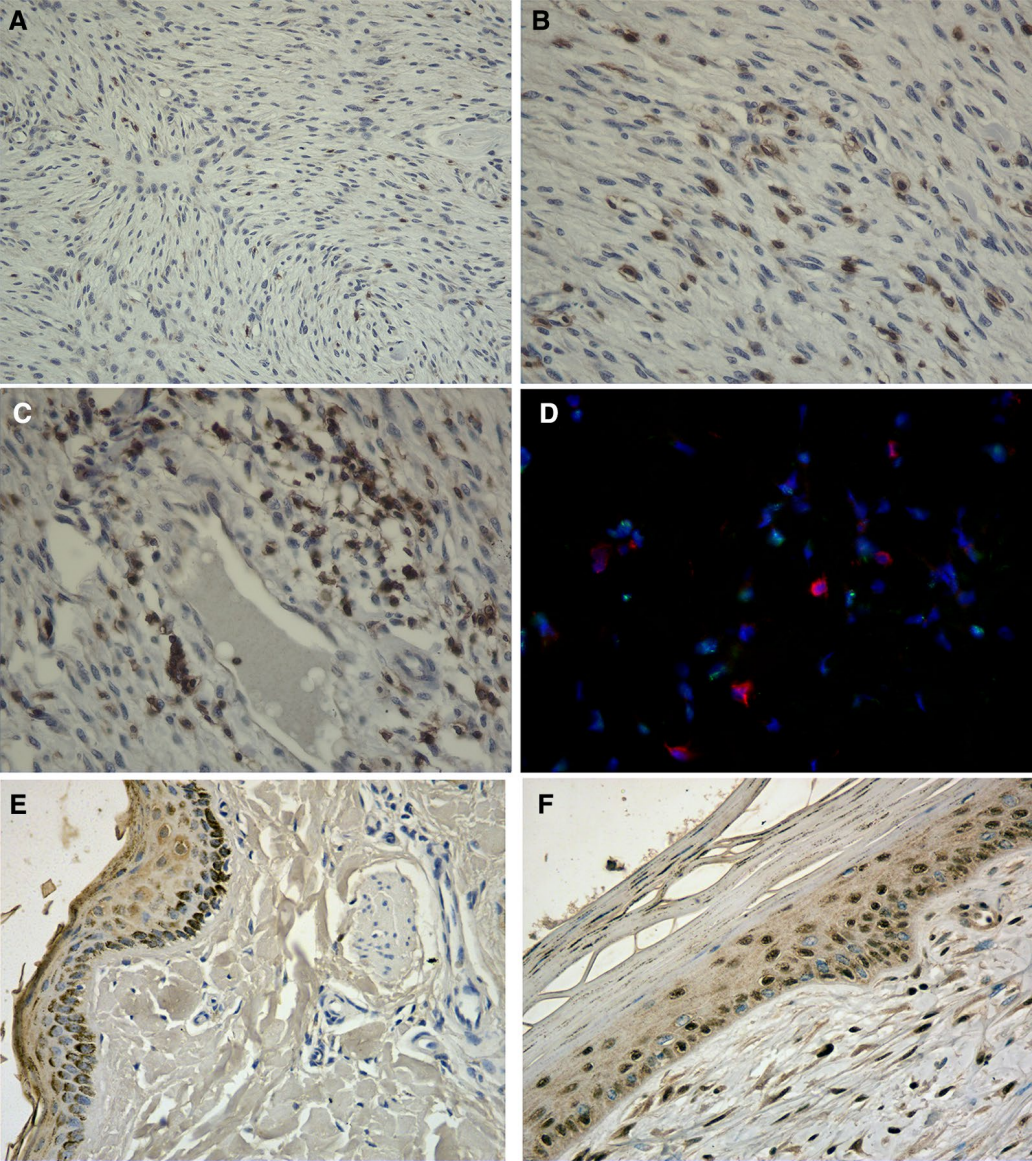
**Immunohistology of sarcoid CD3** ^**+**^ **T-cells and FOXP3 expression. A**–**C** Sections of formalin fixed sarcoid tissue stained using anti-CD3 antibody and peroxidase anti-rabbit antibodies developed with DAB substrate. **A** A low power image (×100) Brown CD3^+^ T-cells are present throughout the tumour. **B** A higher power image (×200) showing the CD3^+^ positive brown staining of cell membranes surrounding a clear cytoplasm and nucleus typical of activated T-cell blasts. **C** An area of dense T-cell infiltration surrounding a blood vessel (×200). **D** Frozen section of sarcoid tissue showing texas-red CD3^+^ T-cells and separate green FITC stained BPV E2 superimposed on blue DAPI nuclear staining. **E** Formalin fixed section of normal equine skin stained with rabbit anti-FOXP3 peroxidase using DAB substrate. The nuclei of cells in the dermis do not express FOXP3, the epidermis shows a diffuse background staining of the keratin, the basal cells contain brown melanin granules but there nuclei are negative for FOXP3 and visible with blue counterstain some FOXP3 positive nuclei are present in the stratum spinosum (arrow). **F** Formalin fixed section of equine sarcoid stained with rabbit anti-FOXP3, the majority of nuclei in the tumour are dark brown indicating they express FOXP3. The FOXP3 staining extends into the nuclei of some basal cells within the epidermis and the stratum spinosum.

Immunohistology of formalin fixed tissues confirmed the presence of many CD3^+^ T-cells within sarcoids (Figures 1A, B and C). Individual T-cells were distributed throughout the tumour, infiltrating between the fibroblasts (Figure 1A), at higher power the T-cells had an activated blast morphology with the presence of distinct membrane staining surrounding an area of cytoplasm with a central nucleus. Clusters of CD3 T-cells were frequently seen adjacent to blood vessels (Figure 1C). Dual staining with rabbit anti-CD3 and donkey anti-rabbit texas red conjugate, along with mouse monoclonal anti-BPV E2 and goat anti-mouse FITC conjugate (Figure 1D) confirmed our earlier observation that BPV E2 is expressed within the nuclei of sarcoid tumour cells and the CD3 T-cells themselves do not express BPV E2 antigen. Staining of fixed tissue samples of normal equine skin using rabbit anti-FOXP3 (ab10563) did not detect any positive cells in the dermis. The keratin layer of the healthy epidermis showed a typical diffuse light brown non-specific background, and the basal cells contained brown melanin granules which make interpretation difficult using DAB substrate. However the basal cell nuclei stained blue with the haematoxylin counterstain indicating they do not express FOXP3 but some scattered cell nuclei in the stratum spinosum were positive (Figure 1E). In comparison the majority of nuclei within the sarcoid tumour mass expressed FOXP3 (Figure 1F). Moreover the nuclei of many basal cells as well as cells in the stratum spinosum of the epidermis overlying the tumour also stained positive for FOXP3 (Figure 1F). Further images of control slides and data for validation of FOXP3 staining using ab10563 (abCam, UK) in horses is provided in Additional files 3, 4, 5, 6, 7 and 8.

**Figure 2:**
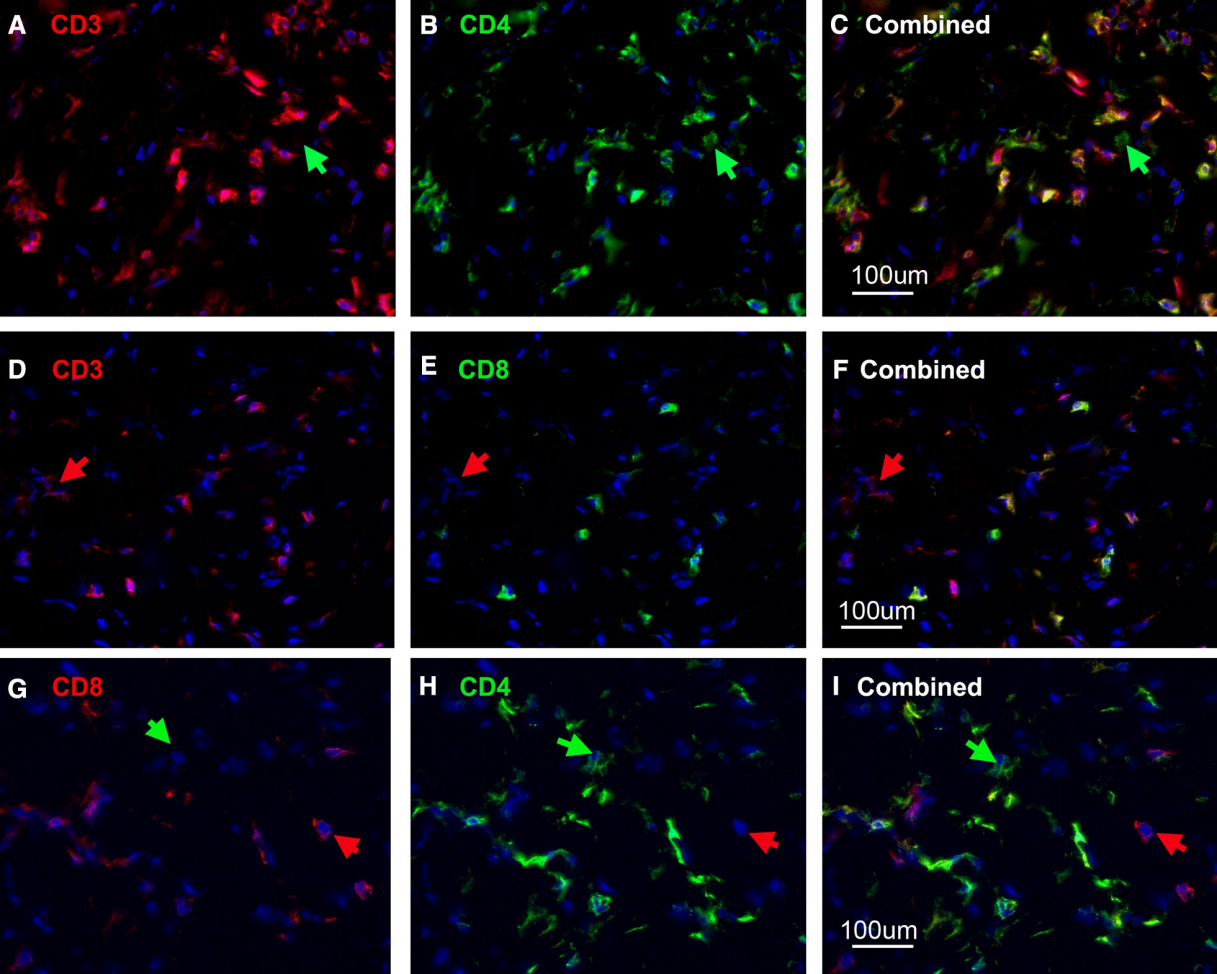
**CD4 and CD8 expression on sarcoid T-cells.** Cryostat sections from a lymphocyte rich area of sarcoid tissue dual stained with different combination of rabbit anti-CD3 and monoclonal mouse anti-equine CD4 or equine CD8α. **A**–**C** Frozen tissue sections dual labelled with (**A**) rabbit anti-CD3 (texas red), (**B**) mouse anti-CD4 (FITC) (**C**) combined image with DAPI nuclear counterstain, most cells express both CD4 and CD3 although some CD4 only cells are present (green arrow). **D**–**F** Frozen tissue sections dual stained with (**D**) rabbit anti-CD3 (texas red), (**E**) mouse anti-CD8 (FITC), (**F**) combined image with DAPI counterstain most cells express both CD3 and CD8 although some CD3 only cells are present (red arrow). **G**–**I** Frozen tissue sections dual labelled with (**G**) monoclonal anti-CD8 TRITC and (**H**) anti-CD4 FITC (**F**) combined image with DAPI nuclear counterstain, the majority of cells are CD4 CD8 dual positive with a few CD8 and CD4 single positive cells (red and green arrows respectively).

To further characterise the phenotype of the tumour infiltrating T-cells, dual staining of sarcoid sections was carried out using rabbit anti-CD3 and donkey anti-rabbit texas red conjugate (Figure 2A) in combination with mouse monoclonal anti-equine CD4 and goat anti-mouse FITC conjugate (Figure 2B). Comparison of the combined images shows that the majority of cells express both CD-antigens. However, there was considerable variation in the intensity of staining, the combined image (Figure 2C) showed a small number of CD3^hi^ CD4^hi^ cells which appear yellow or orange, with the remaining cells appearing green indicating that they express relatively little CD3 in comparison to CD4 although this too appears to be low in intensity on most cells. Occasional CD4^+^ only cells were also present (green arrows Figures 2A and C).

Results from a similar set of images using CD8 in place of CD4 are shown in Figure 2D, E and F; the majority of CD3^+^ cells also express CD8, although most stain only faintly CD8^+^. Several CD3^+^ CD8^−^ cells are also present (red arrow). Dual staining using monoclonal antibodies specific for equine CD4 and CD8 along with isotype specific conjugates (Figures 2G, H and I) confirmed that the majority of T-cells were CD4^lo^ CD8^lo^ dual positive with a small number of CD4^hi^ or CD8^hi^ single positive cells visible (green and red arrows respectively).

**Figure 3:**
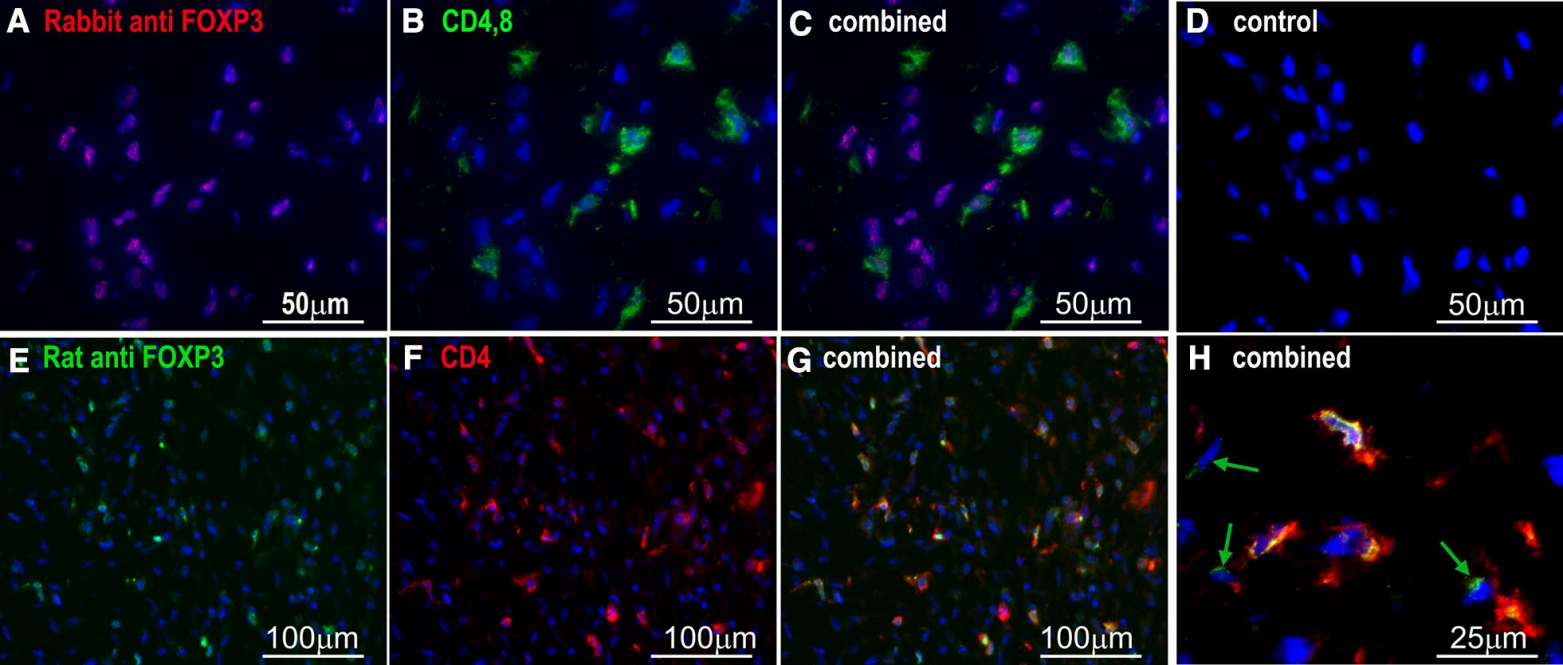
**FOXP3 expression on sarcoid infiltrating T-cells. A** Cryostat sections of sarcoid tissue stained with rabbit anti-FOXP3 (ab 10563) and donkey anti rabbit texas-red with blue DAPI nuclear counterstain. **B** Same section stained using both mouse monoclonal anti-CD4 and monoclonal anti-CD8α with goat anti mouse FITC conjugate. **C** Combined image showing CD4 and CD8 stained T-cells and non T-cells all express FOXP3. **D** Specificity control slide stained with normal rabbit immunoglobulin and isotype matched control mouse monoclonal antibody, DAPI counterstain. **E** Cryostat sections of sarcoid tissue stained with rat anti-FOXP3 (FJK-16s) and donkey anti rat FITC with blue DAPI nuclear counterstain. **F** Same section stained using mouse anti equine CD4 with goat anti mouse TRITC conjugate. **G** Combined image showing both CD4 stained T-cells and non T-cells express FOXP3. **H** Combined image at higher power with CD4-negative FOXP3 positive cells highlighted (arrow).

To determine if the sarcoid infiltrating T-cells had a regulatory phenotype, sections were stained with rabbit anti-FOXP3, in combination with anti-rabbit Texas red (Figure 3A) and a mixture of monoclonal mouse-anti equine CD4 and monoclonal mouse anti-equine CD8 using anti-mouse FITC conjugates (Figure 3B). The results for the combined image confirmed that the CD4^+^ CD8^+^ cells all expressed FOXP3 (Figure 3C); in addition it was noted that many CD4^−^CD8^−^ tumour cells were also FOXP3 positive (Figure 3C) [in agreement with the extensive staining seen in fixed tissues (Figure 3F)]. Control slides, incubated with rabbit and mouse immunoglobulins, were uniformly unstained (Figure 3D).

Rabbit antibody (ab50163) recognises a peptide at the extreme C-terminal of FOXP3 and unexpectedly did not detect any positive cells in an uninflamed lymph node, but did stain numerous cell nuclei in inflamed lymphoid tissue (Additional files 3, 5 and 6). In contrast rat monoclonal anti-mouse Foxp3 which detects an epitope mapped to exon 2 of Foxp3 (FJK-16ss) did stain scattered lymphocytes in resting lymph nodes from horses but not in inflamed lymphoid tissue. Moreover only a few strongly positive cells could be detected in formalin fixed sarcoid tissue stained using FJK-16s, which mostly appeared among lymphoid cell clusters close to blood vessels Additional file 8, although some signs of a weak staining more generally distributed throughout sarcoid tissue were seen, prompting further examination of FOXP3 expression in frozen sections of sarcoid samples stained with FJK-16s. The results confirmed the extensive expression of FOXP3 in sarcoid tissues (Figure 3E) with the strongest expression found in CD4+ cells (Figures 3G and H) although some CD4-ve cells also showed positive staining using this antibody (Figure 3H). The differences in staining patterns of the two anti FOXP3 antibodies can be accounted for if the epitopes recognised by the antibodies in horses, are differentially spliced or removed by post translational proteolytic processing as has been demonstrated to occur in human FOXP3 [38, 39].

**Figure 4:**
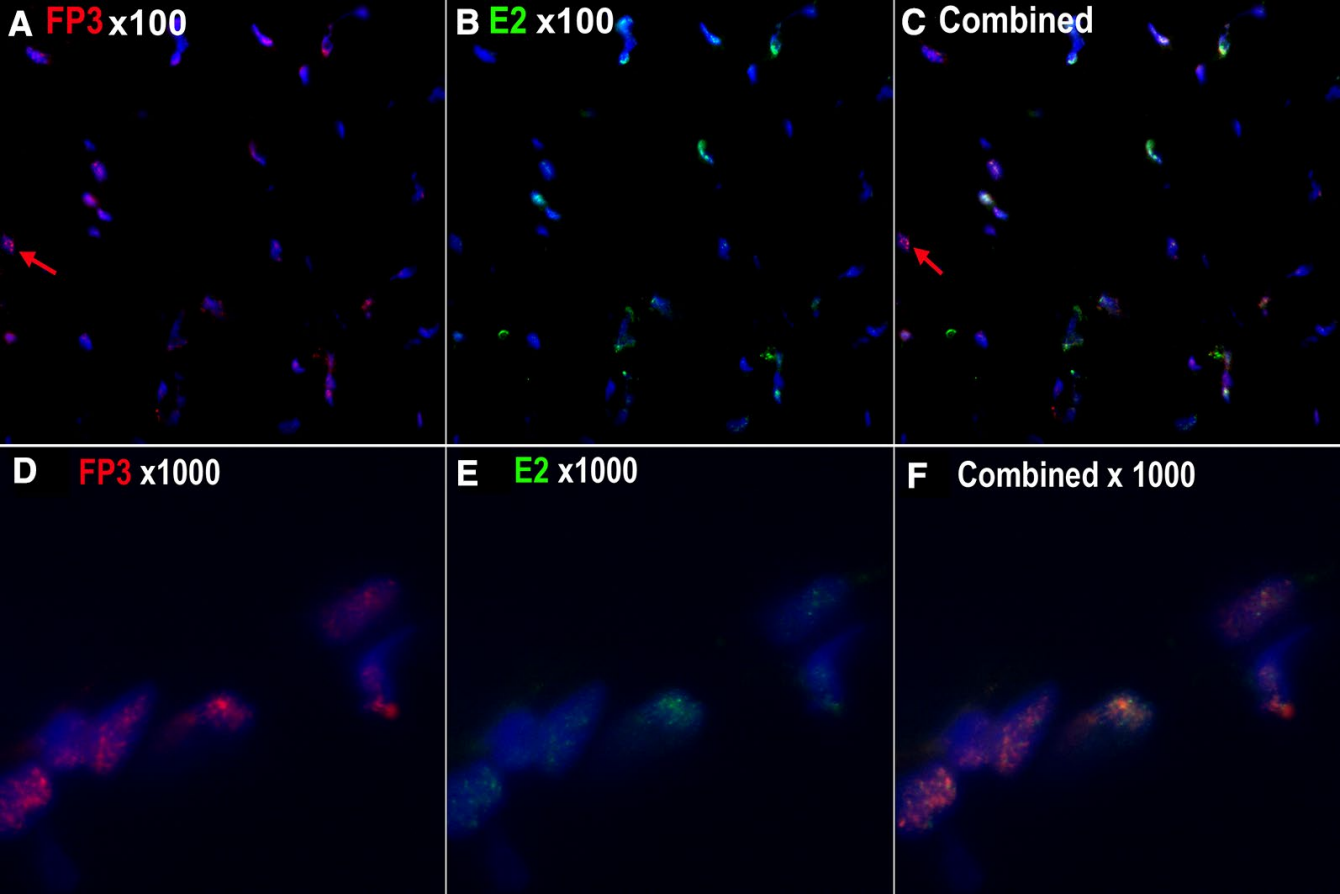
**FOXP3 expression in sarcoid E2 positive tumour cell nuclei. A** Sarcoid tissue stained with rabbit anti-FOXP3 texas-red. **B** Sarcoid tissue stained with mouse monoclonal anti-BPV E2 FITC. **C** dual-stained image with DAPI nuclear counterstain. The majority of nuclei in this image are dual positive tumour cells but some FOXP3 only T-cells are present (red arrow). **D** Higher power image (×1000) of nuclei stained with rabbit anti-FOXP3 texas red. **E** The same nuclei stained with mouse anti-BPV E2 FITC. **F** Higher power image (1000X oil immersion) of dual FOXP3 texas-red and BPV E2 FITC dual-positive sarcoid nuclei, DAPI counterstain.

As staining with anti CD3 and BPV_E2 (Figure 1C) has confirmed our previous finding that T-cells themselves are BPV1 negative, this staining pattern suggests that BPV infected cells must express FOXP3. Dual staining with mouse anti BPVE2 and rabbit anti-FOXP3 confirmed that papillomavirus infected tumour cells co-expressed both antigens in their nuclei (Figure 4).

**Figure 5:**
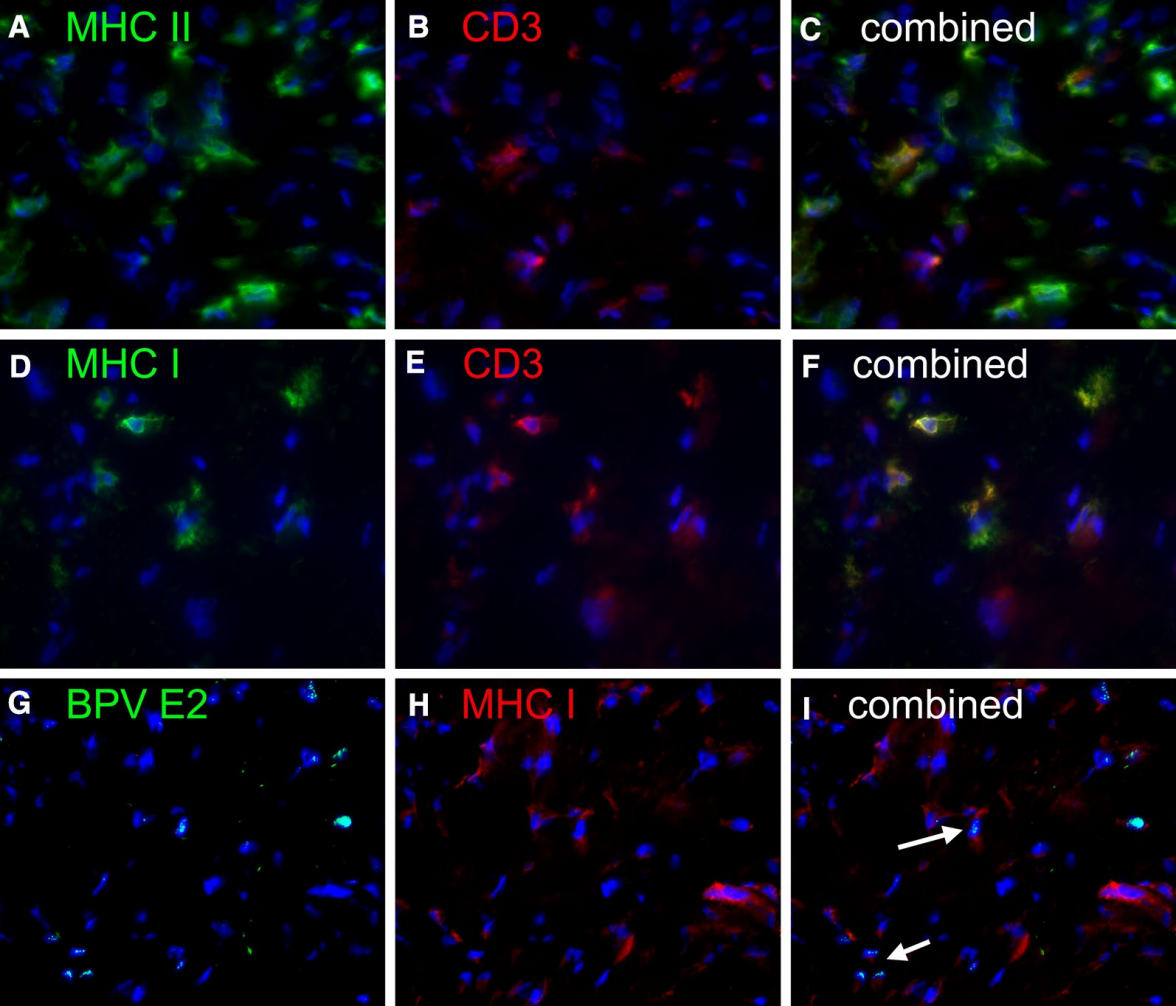
**Sarcoid MHC expression.** Frozen sarcoid tissue stained with (**A**) Mouse monoclonal anti-equine MHC II. Goat anti-mouse FITC. **B** Rabbit anti CD3 donkey anti-rabbit Texas red (**C**) combined image with DAPI counterstain, the majority of MHC II expressing cells are CD3^+^ T-cells. **D** Mouse monoclonal anti-equine MHC I. Goat anti-mouse FITC. **E** Rabbit anti CD3 donkey anti-rabbit Texas red (**F**) combined image with DAPI counterstain the strongly MHC I positive cells are CD3^+^ T-cells. **G** Monoclonal anti-BPV E2 goat anti-mouse IgG1 FITC, (**H**) Monoclonal anti-equine MHC I goat anti mouse IgG2a. **I** Combined image with some possible E2 MHC I dual positive cells (arrow).

Both MHC class I and MHC class II antigens were expressed on a proportion of cells within sarcoids (Figure 5). Many of the MHC II positive cells co-expressed CD3 (Figures 5A, B and C), and given the variable and often weak intensity of CD3 staining it was difficult to determine the extent to which non T-cells expressing MHC II may be present in sarcoid tissues. Similarly MHC class I was also expressed most strongly on CD3 positive cells (Figures 5D, E and F). We next attempted to determine if MHC I was co-expressed on BPV E2 positive tumour cells (Figures 5G, H and I), some co-localisation of MHC I and BPV E2 was detected but is not possible to say whether this was due to membrane surface expression on MHC I on tumour fibroblasts.

### RT-qpcr

**Figure 6:**
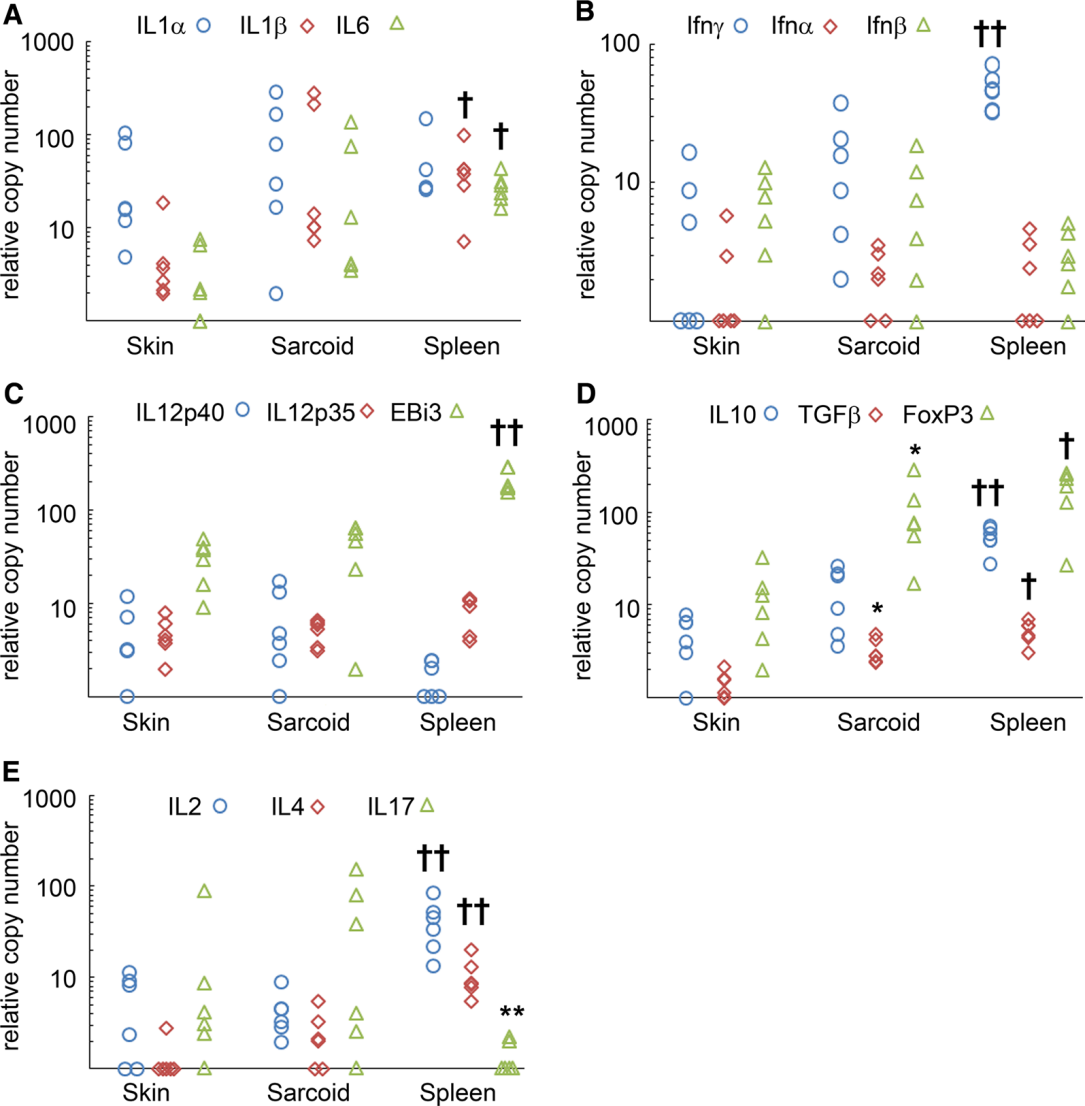
**Quantitiative PCR of cytokine mRNA.** Cytokine mRNA expression as relative copy number. **A** The pro-inflammatory cytokine IL1α did not differ significantly between any of the samples. Both IL1β and IL6 were significantly higher in spleen† compared skin but not to sarcoid, skin and sarcoid did not differ significantly from each other. **B** No significant differences in IFNα or IFNβ levels among the samples were detected. IFNγ was significantly higher in spleen†† compared to either sarcoid or skin, which were not significantly different from each other. **C** There were no significant differences in IL12-p40 or IL12p35 mRNA between the different tissues while EBI3 (IL27βsubunit) was significantly elevated in the spleen†† compared to both skin and sarcoid which were not significantly different from each other. **D** TGFβ and FOXP3 were present in high copy numbers in all tissues there was no difference between sarcoid and spleen but both sarcoid* and spleen† were significantly higher compared to skin. IL10 was significantly higher in spleen†† compared to skin and sarcoid which in turn did not differ significantly from each other. **E** T-cell cytokines IL2 and IL4 were significantly higher in spleen†† compared to skin or sarcoid which were not significantly different from each other. Conversely IL17 was significantly lower in spleen** than either skin or sarcoid.

The results of the cytokine QPCR are shown in Figure 6. Examination of the raw data showed that even though equal amounts of mRNA (50 ng per sample) were reverse transcribed, most cytokine transcripts were lowest in skin, intermediate in sarcoid and highest in spleen reflecting the increasing numbers of lymphocytes found in each tissue. Normalisation of the results using a panel of multiple housekeeping genes reduced the magnitude of this effect but some differences were still apparent.

For the pro-inflammatory cytokines IL1α and IL6, there were no overall significant differences in expression when tested by Kruskal-Wallace ANOVA (KW *p* ≥ 0.05), IL1β did differ between the sample groups (KW *p* = 0.012), but post hoc Mann–Whitey testing showed this was attributable to the spleen having a significantly (*p* < 0.016) higher level of expression compared to skin which did not differ from sarcoid (Figure 6A).

For the interferons (Figure 6B) there were no significant differences in IFNα or IFNβ expression, (KW *p* ≥ 0.05), but IFNγ was significantly different among the groups (KW *p* = 0.0027). Here too post hoc testing showed this was due to the spleen expression levels being significantly higher than either skin or sarcoid (Mann–Whitey *p* < 0.016) which again did not differ from each other.

For the IL12 family members (Figure 6C), neither IL12p40 nor IL12p35 differed between tissues (KW *p* ≥ 0.05). EBI3 expression did show overall significant differences (KW *p* = 0.028), which once again were due to higher levels of expression in spleen compared to either skin or sarcoid tissues (Mann–Whitney <0.016).

The expression of regulatory genes (Figure 6D) showed highly significant differences detected by Kruskal Wallace, with TGFβ *p* = 0.001, IL10 *p* = 0.0017 and FOXP3 *p* = 0.005. Post hoc comparisons showed that both TGFβ and FOXP3 were higher in the sarcoid compared to skin, (TGFβ MW *p* = 0.004, FOXP3 MW *p* = 0.006) making them the only factors found to be significantly higher in sarcoids compared to skin, while the spleen was significantly higher than skin but did not differ from sarcoid. For IL10 the spleen was significantly higher than either skin or sarcoid (MW ≤0.016), but sarcoid and skin did not differ from each other.

The remaining cardinal T-cell cytokines all showed significant differences between tissues (Figure 6E) Kruskall Wallace IL2 *p* = 0.003, IL4 *p* = 0.002 and IL17 *p* = 0.040. In the case of IL2 and IL4, post hoc testing indicated that this was due to the levels of expression being significantly higher in spleen compared to both skin and sarcoid (Mann–Whitney *p* < 0.016), with no difference detected between skin and sarcoid. Although, Kruskall Wallace indicated an overall difference in IL17 expression between the groups (KW *p* = 0.029), post hoc testing using Mann–Whitney and a Bonferroni adjustment of α < 0.016 could not demonstrate which pair of groups differed significantly. However the p value of the Mann–Whitney test between sarcoid and skin was *p* = 0.574 while that of either sarcoid and spleen, or spleen and skin was *p* = 0.020 just short of the Bonferroni corrected value of *p* ≤ 0.016, indicating that the most likely source of the difference detected by Kruskall Wallace is a lower level of IL17 expression in the spleen compared to the other two tissues (Figure 6E).

## Discussion

The epidemiology of equine sarcoids indicates that BPV is transmissible among animals following prolonged close contact [40, 41], implying that it can complete its replication cycle and produce infectious virus within equine hosts. This is supported by observations that equine isolates of BPV1 contain distinct DNA sequence motifs in the long control region [28] and within the BPV1 E5, E2 and L2 gene [9, 29], indicating that equine subtypes of this virus have evolved; yet there has been no convincing demonstration of infectious virus particles within the lesions. Although BPV DNA load and virus early gene transcription correlate with lesion severity confirming the pivotal role for BPV in the pathogenesis of equine sarcoids [42, 43], the persistence of the lesions and apparent absence of an effective anti-viral immune response in equine sarcoids contrasts with the spontaneous immune rejection of BPV associated skin lesions in the majority of infected cattle [3, 16].

Along with the presence of BPV DNA, the aetiology of equine sarcoids can be linked to a number of genetic traits [44]. Genetic associations are particularly evident in relation to MHC genes [45, 46] which together with other genes related to immune response play an important role in host susceptibility to the development of sarcoids [47]. In addition to these host genetic factors acting through increasing the immune susceptibility of horses to BPV in vivo, several features of BPV biology have also been demonstrated to down-regulate immune function in vitro; including the reduction of MHC I expression on the cell surface through its interaction with BPV E5 [30], along with BPV E2 and E7 mediated down-regulation of Toll like receptor 4 [31].

Few studies have been conducted on the immune responses of horses with sarcoids to BPV, but the limited evidence available suggests that there is no naturally occurring antibody to late virus proteins [16, 17], and the induction of neutralizing antibodies by vaccination did not cause reliable regression of the lesions [48, 49]. There is an almost complete lack of information on cell mediated responses to BPV in respect of equine sarcoids. One recent study has demonstrated the up-regulation of FOXP3 and IL10 within sarcoid lesions providing the first evidence for regulatory T-cells as a further mechanism to explain the lack of immune rejection [32], the study also showed IL4 transcription was not increased, but IFNγ was, suggesting that some effector T-cells are also present in sarcoid tissue [32].

In broad agreement with these findings analysis of the data from our QPCR prior to normalisation, (in which equal amounts of total RNA were reverse transcribed Additional file 9), also detected significantly higher levels of FOXP3 and IL10, TGFβ and IFNγ in sarcoids compared to normal skin, with no difference in IL4 and IL2. However, this may simply reflect basal transcription by the higher number of T-cells within sarcoids compared to normal skin, and we therefore included a further set of samples from a lymphoid organ (spleen) along with normalisation to multiple housekeeping genes to gain a better insight into cytokine transcription after taking partial account of the effect of cell number and metabolic activity of different tissues.

Following normalisation to multiple housekeeping genes, the magnitude of the effect seen in respect of IL10 was reduced but TGFβ and FOXP3 transcription in sarcoids remained clearly above that of skin. Additional evidence for the presence of regulatory T-cells in sarcoids was provided by immune-staining which showed that FOXP3 protein is ubiquitously present in the nuclei of sarcoid infiltrating T-cells as well as within the tumour fibroblasts themselves. The high level of TGFβ in all tissues examined is the expected result for the uninflamed tissues (spleen and skin) in which TGFβ provides a tolerance threshold to maintain immune homeostasis and prevent autoimmunity [50] but is somewhat surprising for sarcoids, in which a large number of T-cells with blast morphology are present and would support the conclusion that the T-cells have a regulatory phenotype contributing to the high TGFβ.

Other regulatory cytokine pathways may also contribute to the homeostasis of uninflamed tissues; for example the levels of IL12 family members were biased in favour of EBI3 which along with IL12p35 forms the anti-inflammatory cytokine dimer IL27 compared to the pro-inflammatory IL12p70 formed by combining IL12p35 and IL12p40 [51]; once again the results support the view that sarcoid cytokine expression resembles that of a resting uninflamed tissue. Moreover, following normalisation, there was a lack of evidence to support any increased expression of alpha and beta interferons which could mediate an innate anti-viral response, and normalisation eliminated the differences between normal skin and sarcoid with respect to IFNγ, IL2 and IL4 the key cytokines from Th1 or Th2 differentiated effector T-cells. The exception to this was IL17 which was higher in skin and sarcoid compared to spleen (Figure 6D). IL17 is mainly produced by Th17 cells which are important in host defence against fungi and extracellular bacteria as well as being involved in the pathogenesis of auto-immune and chronic inflammatory diseases; but increasing evidence supports a role for Th17 cells in immune regulation [52]. Interestingly elevated IL17 production by CD4^+^ T-cells has recently been demonstrated in HPV-associated cervical intraepithelial neoplasia [53]. Increased IL17 expression by T-cells also occurs in the hyperplastic skin lesions of E7 transgenic mice in which the HPV16 E7 gene is expressed under control of the K14 promoter in basal epithelial cells [53]. The authors went on to demonstrate that IL17 T-cells suppressed immunological clearance of HPV E7 transgenic epithelial grafts in mice [53], although whether IL17 producing T-cells could have a similar suppressive role in equine sarcoids cannot be easily determined.

In human papillomavirus associated malignancies, there is a strong association between the occurrence of regulatory T-cells within the lesions, disease progression and treatment outcome [54–57]. This paper provides further evidence of an important role of regulatory T-cells in maintaining the papilloma virus infection of equine sarcoids; in which almost all infiltrating T-cells stained positive for nuclear FOXP3, the key transcription factor for T-reg cells of mammals including horses [58–60]. The co-expression of FOXP3 and BPV E2 within the virus transformed cells adds an extra dimension to its potential role in the pathogenesis of sarcoids. Several types of non-lymphoid tumour cells are reported to have up-regulated FOXP3 expression in the cell nucleus, including pancreas [61], melanoma [62], and epithelial cells of papilloma virus associated cervical cancer [63]. In vitro experiments have shown the proliferation of activated T-cells to be inhibited in the presence FOXP3-expressing pancreatic cancer cell lines, indicating that tumour cells themselves can directly contribute to an immune-regulatory microenvironment; although the mechanism by which this was mediated was not defined [61]. How BPV genes co-expressed with FOXP3 interact and what role (if any) that FOXP3 may have in inducing regulatory pathways within sarcoid fibroblasts remains to be investigated.

Immunofluorescence staining of sarcoid infiltrating CD3+ T-cells indicated that in addition to FOXP3 the majority were CD4^+^CD8^+^ dual positive cells and expressed MHC class I and class II on their surface. CD4^+^CD8^+^ dual positive T-cells are most often associated with immature T-cells within the thymus, but in humans extra-thymic CD4^+^CD8^+^ dual positive T-cells are also found in several chronic pathological inflammatory conditions or chronic virus infections and notably CD4^+^CD8^+^ dual positive lymphocytes are present in normal human skin [64, 65]. In mice, a population of CD4^+^ CD8^αα^ dual positive T-cells that have a regulatory function are present in the intestinal epithelial compartment [66]. In primates and pigs CD4^+^CD8^+^ dual positive cells represent a population of memory cells derived from MHC class II restricted CD4^+^ precursors which express CD8^α^ following activation [67]. Most recently a population of CD4^+^ CD8^+^ CD25^+^ FOXP3^+^ induced T-reg cells have been identified in pigs infected with Porcine reproductive and respiratory syndrome virus where their numbers correlated with viraemia [68] indicating that they too have an immune-regulatory function. The consensus of evidence favours extra-thymic dual positive T-cells being a result of chronic activation and having a regulatory phenotype which also fits well with the observed lack of effector cytokine expression seen in sarcoids.

It remains unclear what antigens the T-cells in sarcoids may be responding to and how that antigen is presented. The majority of MHC class II cells in the tumour appear to be T-cells which are not usually considered to be professional antigen presenting cells. Nevertheless MHC class II is expressed on activated CD3 T-cells in most mammals including horses and humans, but not in mice [69, 70]. However, the outcome of antigen presentation between T-cells is usually one of anergy or apoptosis rather than activation and their role in immune responses is thought to be largely one of regulation [70] providing a further potential avenue of immune regulation in sarcoids.

Overall the evidence indicates that equine sarcoids provide a regulatory immune microenvironment in which the replication of BPV infected fibroblast proceeds unchecked by the immune system. This insight can alter many of the interpretations placed on prior experiments investigating the aetiology and pathogenesis of sarcoids: for example the reports of successful transmission of sarcoids by grafting tumour explants [13] may now be interpreted as a failure to reject the allografted tissue due to its capacity to establish a tolerogenic cytokine microenvironment, as opposed to transmission via de novo virus infection. These insights into the nature of sarcoid pathogenesis will also inform our interpretation of current and future treatment strategies.

## Additional files

10.1186/s13567-016-0339-8 **Table of primers and probes.** Additional material including all sequences for housekeeping and cytokine gene primer and probe sets, along with the efficiency for each PCR reaction.
10.1186/s13567-016-0339-8 **Table of statistical results for normalised cytokine mRNA analysis.** The individual p values for all statistical comparisons between cytokine mRNA relative copy-number in each tissue are tabulated.
10.1186/s13567-016-0339-8 **Data for validation data for FOXP3 staining with ab10563.** (abCam UK). Peptide specificity of the antibody and additional control slides of various tissues are presented.
10.1186/s13567-016-0339-8 **Images of FOXP3 staining in normal equine skin using (ab10563 abCam, UK), (FJK-16s eBioscience) and normal rabbit serum.** Images **A** and **D**, control sections with normal rabbit serum show some background staining of cytoplasm in sebaceous glands (this type of background is typical of many antibodies). Also brown melanin granules are visible in the basal epidermis but nuclei are unstained (blue colour). Sections **B** and **E** show normal equine skin stained with Rat anti mouse FoxP3 (FJK-16s). The sections have the same background brown coloration of sebaceous gland cytoplasm as well as melanin containing hair shafts and basal cells but again there is no clear staining of nuclei. Sections **C** and **F** sow equine skin stained with Rabbit anti FOXP3 antibody (Ab 10563) once again showing the same background brown coloration of sebaceous gland cytoplasm, but in addition clear FOXP3 staining of the nuclei of sebaceous glands and nuclei of some cells in the stratum spinosum as well as the nuciei of a proportion of cells in some sections of hair follicle.
10.1186/s13567-016-0339-8 **Inflamed equine skin stained with (ab10563 abCam, UK).** A section of inflamed equine skin from a case of chronic chorioptic mange on the lower limb of a horse stained with Rabbit anti Human FOXP3 (ab10563 abCam UK). When stained with this antibody inflamed skin reveals more FOXP3 positive cells in the dermis and epidermis compared to normal skin.
10.1186/s13567-016-0339-8 **Sections of inflamed and uniflamed equine lymph node stained with (ab10563 abCam, UK) or (FJK-16s eBioscience).** **A** The T-cell areas in an uninflamed mesenteric lymph node revealed no FOXP3 positive cells using Rabbit anti FOXP3 ( Ab ab10563 abCam UK) **B** The same tissue stained with rat anti-FoxP3 (FJK-16s) demonstrating scattered positive staining of lymphocytes. This contrasts with a section of inflamed lymph node in which rabbit anti FOXP3 (ab10563) stains many cells in the follicular and inter-follicular areas **C**, yet in this tissue FJK-16s detected no positive cells **D**.
10.1186/s13567-016-0339-8 **Sections of normal equine small intestine stained with (ab10563 abCam, UK) or (FJK-16s eBioscience).** **A** rabbit anti FOXP3 and **B** Rat anti FoxP3 both antibodies detected nuclear staining of populations of cells in the lamina propria of equine small intestine.
10.1186/s13567-016-0339-8 **Sections of equine sarcoid stained with rat anti FoxP3.** **A** Formalin fixed equine sarcoid stained with rat anti FoxP3 (FJK-16s eBioscience) positive stained cell were detected particularly in areas adjacent to blood vessels. **B** no staining was observed using isotype control antibody.
10.1186/s13567-016-0339-8 **Table of statistical results for raw PCR data from cytokine mRNA analysis.** The individual *p* values for all statistical comparisons between cytokine mRNA QPCR_CT number in each tissue are tabulated.

## References

[1] Gottschling M, Goker M, Stamatakis A, Bininda-Emonds OR, Nindl I, Bravo IG (2011). Quantifying the phylodynamic forces driving papillomavirus evolution. Mol Biol Evol.

[2] Marais HJ, Page PC (2011). Treatment of equine sarcoid in seven Cape mountain zebra (*Equus zebra* zebra). J Wild Dis.

[3] Jarret WHF, G K (1985). The natural history of bovine papillomavirus infections. advances in viral oncology.

[4] Nasir L, McFarlane ST, Torrontegui BO, Reid SW (1997). Screening for bovine papillomavirus in peripheral blood cells of donkeys with and without sarcoids. Res Vet Sci.

[5] Martens A, De Moor A, Demeulemeester J, Ducatelle R (2000). Histopathological characteristics of five clinical types of equine sarcoid. Res Vet Sci.

[6] Sironi G, Caniatti M, Scanziani E (1990). Immunohistochemical detection of papillomavirus structural antigens in animal hyperplastic and neoplastic epithelial lesions. Zentralbl Veterinarmed A.

[7] Sundberg JP, Junge RE, Lancaster WD (1984). Immunoperoxidase localization of papillomaviruses in hyperplastic and neoplastic epithelial lesions of animals. Am J Vet Res.

[8] Tajima M, Gordon DE, Olson C (1968). Electron microscopy of bovine papilloma and deer fibroma viruses. Am J Vet Res.

[9] Wilson AD, Armstrong EL, Gofton RG, Mason J, De Toit N, Day MJ (2013). Characterisation of early and late bovine papillomavirus protein expression in equine sarcoids. Vet Microbiol.

[10] Olson C, Cook RH (1951). Cutaneous sarcoma-like lesions of the horse caused by the agent of bovine papilloma. Proc Soc Exp Biol Med.

[11] Ragland WL, Spencer GR (1969). Attempts to relate bovine papilloma virus to the cause of equine sarcoid: equidae inoculated intradermally with bovine papilloma virus. Am J Vet Res.

[12] Lee KP, Olson C (1968). Response of calves to intravenous and repeated intradermal inoculation of bovine papilloma virus. Am J Vet Res.

[13] Voss JL (1969). Transmission of equine sarcoid. Am J Vet Res.

[14] Barthold SW, Olson C (1974). Fibroma regression in relation to antibody and challenge immunity to bovine papilloma virus. Cancer Res.

[15] Bergvall KE (2013). Sarcoids. Vet Clin North Am Equine Pract.

[16] Ragland WL, Spencer GR (1968). Attempts to relate bovine papilloma virus to the cause of equine sarcoid: immunity to bovine papilloma virus. Am J Vet Res.

[17] Segre D, Olson C, Hoerlein AB (1955). Neutralization of bovine papilloma virus with serums from cattle and horses with experimental papillomas. Am J Vet Res.

[18] Amtmann E, Muller H, Sauer G (1980). Equine connective tissue tumors contain unintegrated bovine papilloma virus DNA. J Virol.

[19] Brandt S, Tober R, Corteggio A, Burger S, Sabitzer S, Walter I, Kainzbauer C, Steinborn R, Nasir L, Borzacchiello G (2011). BPV-1 infection is not confined to the dermis but also involves the epidermis of equine sarcoids. Vet Microbiol.

[20] Borzacchiello G, Russo V, Della Salda L, Roperto S, Roperto F (2008). Expression of platelet-derived growth factor-beta receptor and bovine papillomavirus E5 and E7 oncoproteins in equine sarcoid. J Comp Pathol.

[21] Carr EA, Theon AP, Madewell BR, Hitchcock ME, Schlegel R, Schiller JT (2001). Expression of a transforming gene (E5) of bovine papillomavirus in sarcoids obtained from horses. Am J Vet Res.

[22] Nasir L, Reid SWJ (1999). Bovine papillomaviral gene expression in equine sarcoid tumours. Virus Res.

[23] Jelinek F, Tachezy R (2005). Cutaneous papillomatosis in cattle. J Comp Pathol.

[24] Lory S, von Tscharner C, Marti E, Bestetti G, Grimm S, Waldvogel A (1993). In situ hybridisation of equine sarcoids with bovine papilloma virus. Vet Rec.

[25] Wobeser BK, Hill JE, Jackson ML, Kidney BA, Mayer MN, Townsend HGG, Allen AL (2012). Localization of Bovine papillomavirus in equine sarcoids and inflammatory skin conditions of horses using laser microdissection and two forms of DNA amplification. J Vet Diagn Invest.

[26] Gaynor AM, Zhu KW, Cruz FN, Affolter VK, Pesavento PA (2016). Localization of bovine papillomavirus nucleic acid in equine sarcoids. Vet Pathol.

[27] Brandt S, Haralambus R, Shafti-Keramat S, Steinborn R, Stanek C, Kirnbauer R (2008). A subset of equine sarcoids harbours BPV-1 DNA in a complex with L1 major capsid protein. Virology.

[28] Trewby H, Ayele G, Borzacchiello G, Brandt S, Campo MS, Del Fava C, Marais J, Leonardi L, Vanselow B, Biek R, Nasir L (2014). Analysis of the long control region of bovine papillomavirus type 1 associated with sarcoids in equine hosts indicates multiple cross-species transmission events and phylogeographic structure. J Gen Virol.

[29] Chambers G, Ellsmore VA, O’Brien PM, Reid SW, Love S, Campo MS, Nasir L (2003). Sequence variants of bovine papillomavirus E5 detected in equine sarcoids. Virus Res.

[30] Marchetti B, Gault EA, Cortese MS, Yuan ZQ, Ellis SA, Nasir L, Campo MS (2009). Bovine papillomavirus type 1 oncoprotein E5 inhibits equine MHC class I and interacts with equine MHC I heavy chain. J Gen Virol.

[31] Yuan ZQ, Bennett L, Campo MS, Nasir L (2010). Bovine papillomavirus type 1 E2 and E7 proteins down-regulate Toll Like Receptor 4 (TLR4) expression in equine fibroblasts. Virus Res.

[32] Mahlmann K, Hamza E, Marti E, Dolf G, Klukowska J, Gerber V, Koch C (2014). Increased FOXP3 expression in tumour-associated tissues of horses affected with equine sarcoid disease. Vet J.

[33] Kurg R, Parik J, Juronen E, Sedman T, Abroi A, Liiv I, Langel U, Ustav M (1999). Effect of bovine papillomavirus E2 protein-specific monoclonal antibodies on papillomavirus DNA replication. J Virol.

[34] Pittaway CE, Lawson AL, Coles GC, Wilson AD (2014). Systemic and mucosal IgE antibody responses of horses to infection with Anoplocephala perfoliata. Vet Parasitol.

[35] Primer3web version 4.0.0. www.bioinfo.ut.ee

[36] mFold www.bioinfo.rpi.edu

[37] Vandesompele J, DePreter K, Pattyn F, Poppe B, Van Roy N, DePaepe A, Speleman F (2002). Accurate normalization of real-time quantitative RT-PCR data by geometric averaging of multiple internal control genes. Genome Biol.

[38] de Zoeten EF, Lee I, Wang L, Chen C, Ge G, Wells AD, Hancock WW, Ozkaynak E (2009). Foxp3 processing by proprotein convertases and control of regulatory T cell function. J Biol Chem.

[39] Ziegler SF (2006). FOXP3: of mice and men. Annu Rev Immunol.

[40] Reid SW, Gettinby G, Fowler JN, Ikin P (1994). Epidemiological observations on sarcoids in a population of donkeys (*Equus asinus*). Vet Rec.

[41] Ragland WL, Keown GH, Gorham JR (1966). An epizootic of equine sarcoid. Nature.

[42] Haralambus R, Burgstaller J, Klukowska-Rotzler J, Steinborn R, Buchinger S, Gerber V, Brandt S (2010). Intralesional bovine papillomavirus DNA loads reflect severity of equine sarcoid disease. Equine Vet J.

[43] Bogaert L, Van Poucke M, De Baere C, Dewulf J, Peelman L, Ducatelle R, Gasthuys F, Martens A (2007). Bovine papillomavirus load and mRNA expression, cell proliferation and p53 expression in four clinical types of equine sarcoid. J Gen Virol.

[44] Christen G, Gerber V, Dolf G, Burger D, Koch C (2014). Inheritance of equine sarcoid disease in Franches-Montagnes horses. Vet J.

[45] Lazary S, Marti M, Szalai G, Gaillard C, Gerber H (1994). Studies on the frequency and associations of equine leukocyte antigens in sarcoid and summer dermatitis. Anim Genet.

[46] Brostrom H (1995). Equine sarcoids. A clinical and epidemiological study in relation to equine leucocyte antigens (ELA). Acta Vet Scand.

[47] Jandova V, Klukowska-Rotzler J, Dolf G, Janda J, Roosje P, Marti E, Koch C, Gerber V, Swinburne J (2012). Whole genome scan identifies several chromosomal regions linked to equine sarcoids. Schweiz Arch Tierheilkd.

[48] Mattil-Fritz S, Scharner D, Piuko K, Thones N, Gissmann L, Muller H, Muller M (2008). Immunotherapy of equine sarcoid: dose-escalation trial for the use of chimeric papillomavirus-like particles. J Gen Virol.

[49] Ashrafi GH, Piuko K, Burden F, Yuan Z, Gault EA, Muller M, Trawford A, Reid SWJ, Nasir L, Campo MS (2008). Vaccination of sarcoid-bearing donkeys with chimeric virus-like particles of bovine papillomavirus type 1. J Gen Virol.

[50] Germain RN (2012). Maintaining system homeostasis: the third law of Newtonian immunology. Nat Immunol.

[51] Vignali DA, Kuchroo VK (2012). IL-12 family cytokines: immunological playmakers. Nat Immunol.

[52] Korn T, Bettelli E, Oukka M, Kuchroo VK (2009). IL-17 and Th17 cells. Annu Rev Immunol.

[53] Gosmann C, Mattarollo SR, Bridge JA, Frazer IH, Blumenthal A (2014). IL-17 suppresses immune effector functions in human papillomavirus-associated epithelial hyperplasia. J Immunol.

[54] Cao Y, Zhao J, Lei Z, Shen S, Liu C, Li D, Liu J, Shen GX, Zhang GM, Feng ZH, Huang B (2008). Local accumulation of FOXP3+ regulatory T cells: evidence for an immune evasion mechanism in patients with large condylomata acuminata. J Immunol.

[55] Nasman A, Romanitan M, Nordfors C, Grun N, Johansson H, Hammarstedt L, Marklund L, Munck-Wikland E, Dalianis T, Ramqvist T (2012). Tumor infiltrating CD8+ and Foxp3+ lymphocytes correlate to clinical outcome and human papillomavirus (HPV) status in tonsillar cancer. PLoS One.

[56] Molling JW, de Gruijl TD, Glim J, Moreno M, Rozendaal L, Meijer CJ, van den Eertwegh AJ, Scheper RJ, von Blomberg ME, Bontkes HJ (2007). CD4(+)CD25hi regulatory T-cell frequency correlates with persistence of human papillomavirus type 16 and T helper cell responses in patients with cervical intraepithelial neoplasia. Int J Cancer.

[57] van Esch EM, van Poelgeest MI, Kouwenberg S, Osse EM, Trimbos JB, Fleuren GJ, Jordanova ES, van der Burg SH (2015). Expression of coinhibitory receptors on T cells in the microenvironment of usual vulvar intraepithelial neoplasia is related to proinflammatory effector T cells and an increased recurrence-free survival. Int J Cancer.

[58] Hamza E, Gerber V, Steinbach F, Marti E (2011). Equine CD4(+) CD25(high) T cells exhibit regulatory activity by close contact and cytokine-dependent mechanisms in vitro. Immunology.

[59] Sakaguchi S, Yamaguchi T, Nomura T, Ono M (2008). Regulatory T cells and immune tolerance. Cell.

[60] Ramsdell F, Ziegler SF (2014). FOXP3 and scurfy: how it all began. Nat Rev Immunol.

[61] Hinz S, Pagerols-Raluy L, Oberg HH, Ammerpohl O, Grussel S, Sipos B, Grutzmann R, Pilarsky C, Ungefroren H Saeger HD, Kloppel G, Kabelitz D, Kalthoff H (2007). Foxp3 expression in pancreatic carcinoma cells as a novel mechanism of immune evasion in cancer. Cancer Res.

[62] Ebert LM, Tan BS, Browning J, Svobodova S, Russell SE, Kirkpatrick N, Gedye C, Moss D, Ng SP, MacGregor D, Davis ID, Cebon J, Chen W (2008). The regulatory T cell-associated transcription factor FoxP3 is expressed by tumor cells. Cancer Res.

[63] Zeng C, Yao Y, Jie W, Zhang M, Hu X, Zhao Y, Wang S, Yin J, Song Y (2013). Up-regulation of Foxp3 participates in progression of cervical cancer. Cancer Immunol Immunother.

[64] Parel Y, Aurrand-Lions M, Scheja A, Dayer JM, Roosnek E, Chizzolini C (2007). Presence of CD4+ CD8+ double-positive T cells with very high interleukin-4 production potential in lesional skin of patients with systemic sclerosis. Arthritis Rheum.

[65] Nascimbeni M, Pol S, Saunier B (2011). Distinct CD4+ CD8+ double-positive T cells in the blood and liver of patients during chronic hepatitis B and C. PLoS One.

[66] Das G, Augustine MM, Das J, Bottomly K, Ray P, Ray A (2003). An important regulatory role for CD4 + CD8 alpha alpha T cells in the intestinal epithelial layer in the prevention of inflammatory bowel disease. Proc Natl Acad Sci U S A.

[67] Zuckermann FA (1999). Extrathymic CD4/CD8 double positive T cells. Vet Immunol Immunopathol.

[68] Silva-Campa E, Mata-Haro V, Mateu E, Hernandez J (2012). Porcine reproductive and respiratory syndrome virus induces CD4+ CD8+ CD25+ Foxp3+ regulatory T cells (Tregs). Virology.

[69] Bendali-Ahcene S, Cadore JL, Fontaine M, Monier JC (1997). Anti-alpha chain monoclonal antibodies of equine MHC class-II antigens: applications to equine infectious anaemia. Res Vet Sci.

[70] Holling TM, Schooten E, van Den Elsen PJ (2004). Function and regulation of MHC class II molecules in T-lymphocytes: of mice and men. Hum Immunol.

