# Supplementary material for: Both tumour cells and infiltrating T-cells in equine sarcoids express FOXP3 associated with an immune-supressed cytokine microenvironment
Source: Vet Res. 2016 May 9;47:55. doi: 10.1186/s13567-016-0339-8 (PMC4862206; doi:10.1186/s13567-016-0339-8)
Supplement: Supplementary file 1 — 10.1186/s13567-016-0339-8 Table of primers and probes. Additional material including all sequences for housekeeping and cytokine gene primer and probe sets, along with the efficiency for each PCR reaction. [file 13567_2016_339_MOESM1_ESM.docx]

| Gene |  | Primer sequence | Probe sequence | Efficiency % |
| --- | --- | --- | --- | --- |
| Actb | Fwd | CCAGCACGATGAAGATCA | TCCGTATGGATCGGCGGC | 98% r = 1.0 |
|  | Rev | CTGGAAGGTGGACAATGA |  |  |
| B2M | Fwd | TTTACTCACGTCACCCAGCA | TGGAAAGCCAAATTTCCTGAACTG | 98.8% r = 1.0 |
|  | Rev | AATCTCAGGCGGATGGAAC |  |  |
| GAPDH | Fwd | GGAAGCTCACTGGCATGG | CTTCCGTGTCCCCACCCCTAAC | 95.3% r = 1.0 |
|  | Rev | CGTATTTGGCAGCTTTCTCC |  |  |
| HPRT1 | Fwd | AAGATGGTCAAGGTCGCAAG | TGCTGGTGAAAAGGACCCCTCG | 96.6% r = 0.999 |
|  | Rev | TCAAATCCAACAAAGTCTGGTC |  |  |
| RPL32 | Fwd | AAATTCATTCGGCACCAGTC | CTGGCGGAAACCCAGAGGCA | 98.1% r = 0.999 |
|  | Rev | GCATCAATATCTGGCCCTTG |  |  |
| TUBA1 | Fwd | ATTGGGCAAGGGAGATCATT | CTGGACCGGATCCGCAAGCT | 92.7% r = 0.999 |
|  | Rev | AAGCTGTGAAACACCAGGAAG |  |  |
| UBB | Fwd | TCAGAGGTGGGATGCAGATT | CACCCTGGAGGTGGAGCCCA | 99.9% r = 0.999 |
|  | Rev | TGGATCTTGGCCTTCACATT |  |  |
| Eq_IL1a | Fwd | TGTGAGTGCCCAAAATGAAG | TTTTGGGTGTGTCAGGCATCTCC | 99.6% r = 0.997 |
|  | Rev | CCAGAAGAAGAGGAGGTTGGT |  |  |
| Eq_IL1b | Fwd | GCATCCAGCTTCAATTCTCC | CCTGTGAGCAGGGAACGGGTATCT | 101.1% r = 0.997 |
|  | Rev | GGCTCCTCAAGTCATCATCC |  |  |
| Eq_IL2 | Fwd | GGCTTGCATCGCACTAACTC | TGCAGTCCTTGCAAACAGTGCACC | 98% r = 0.998 |
|  | Rev | GTTGTTGCTGTGTTTCCCTCT |  |  |
| Eq_IL4 | Fwd | CTGCAAAGGTGCTTCAACAG | CAGGTCCCGTTTGCCATGCC | 99.8% r = 0.99  107% |
|  | Rev | TGTGCTCTTCTTGGCTTCATT |  |  |
| Eq_IL6 | Fwd | CCTGGTGATGGCTACTGCTT | CCCACCCCACTACCCCTGGG | 99.4% r = 1.0 |
|  | Rev | CAGAGATTTTGCCGAGGATG |  |  |
| Eq_IL10 | Fwd | CGATTTCTGCCCTGTGAAA | TGGAGCAGGTGAAGAGTGCCTTCA | 95.8% r = 1.0 |
|  | Rev | CACTCATGGCTTTGTAGACACCT |  |  |
| Eq_IL12p35 | Fwd | AATGTTCCAGTGCCTCAACC | TGAGGACCGTCAGCAACACGC | 98.2% r = 0.990 |
|  | Rev | TGCTGCTCTTGTCTTTTGTGA |  |  |
| Eq_IL12p40 | Fwd | AGATCGTGGTGGATGCTGTT | TGAAAACTACACCAGCGGCTTCTTCA | 98.8% r = 0.998 |
|  | Rev | TGGTTTGATGATGTCCCTGA |  |  |
| Eq_EBI3 (IL27b) | Fwd | CGTTGCTGGGCTTCTAGGT | CTGGACGCTGCCACCTGCTG | 100.01% r = 0.999 |
|  | Rev) | AGCCTGTACGTGGCAATGA |  |  |
| Eq_IL17 | Fwd | CGTAAGGCGGGAATAGTAA | CGGAATGCCCGAACACTGGG | 98.9% r = 1.0 |
|  | Rev | GTGGAGCGGTTGTGGTAATC |  |  |
| Eq_IFNg | Fwd | CCTAACTCTCTCCGAAACAATGAA | TCGCACACAGCTGAAAAGCCAA | 96.5% r = 1.0 |
|  | Rev | GCAGTAATAGGTAGAAGAACCCAAA |  |  |
| Eq_FOXP3 | Fwd | CAGTGTGGAATGGGTGTCC | CCTTCCCAAGCCCCAGTGCA | 99.4% r = 0.999 |
|  | Rev | AAAGGGTGCTGTCTTTCCTG |  |  |
| Eq_TGFb | Fwd | GGACTACTACGCCAAGGAGGT | CACCCGCGTGCTAATGGTGGA | 96.6% r = 1.0 |
|  | Rev | AGCCGGTCTCCACAGTCTTAT |  |  |
| Eq_TNFa | Fwd | CCTCAGCCTCTTCTCCTTCC | TTGTCGCAGGAGCCACCACG | 100.4% r = 1.0 |
|  | Rev | AAGGCATTCGGTAACTGCTC |  |  |
| Eq_IFNa | Fwd | TGCCTGAAGGACAGAAATGA | TGACGGCAACCAGTTCCGGA | 99.8% r = 0.998 |
|  | Rev | TGTGCTGAAGAGGTGGAAGA |  |  |
| Eq_IFNb | Fwd | AATGGCCCTCCTGCTGT | TCTCCACCACGGCTCTTTCTGTGAA | 99.5% r = 0.999 |
|  | Rev | CATGCTGAATTGCTGCTTCT |  |  |
