# Supplementary material for: Both tumour cells and infiltrating T-cells in equine sarcoids express FOXP3 associated with an immune-supressed cytokine microenvironment
Source: Vet Res. 2016 May 9;47:55. doi: 10.1186/s13567-016-0339-8 (PMC4862206; doi:10.1186/s13567-016-0339-8)
Supplement: Supplementary file 2 — 10.1186/s13567-016-0339-8 Table of statistical results for normalised cytokine mRNA analysis. The individual p values for all statistical comparisons between cytokine mRNA relative copy-number in each tissue are tabulated. [file 13567_2016_339_MOESM2_ESM.docx]

**Table of statistical analysis of results for normalised cytokine mRNA data**

**Kruskal Wallace Mann Whitney Mann Whitney Mann Whitney**

**α = 0.05 α = 0.016 α = 0.016 α = 0.016**

**All tissues Skin vs sarcoid Sarc vs Spleen Spleen Vs Skin**

IL1_ alpha *p* = 0.413 *p* = 0.337 *p* = 0.631 *p* = 0.200

Il1_beta *p* = 0.014 *p* = 0.025 *p* = 0.748 spl>sk *p* = 0.006

IL6 *p* = 0.012 *p* = 0.054 *p* = 0.337 spl>sk *p* = 0.004

Ifn_gamma *p* = 0.0031 *p* = 0.107 spl> sarc *p* = 0.010 spl>sk *p* = 0.004

Ifn_alpha *P* = 0.316 *p* = 0.494 *p* = 0.86 *p* = 0.721

Ifn_beta *P* = 0.271 *p* = 0.936 *p* = 0.297 *p* = 0.092

IL12p40 *p* = 0.57 *p* = 0.574 *p* = 0.042 *p* = 0.028

IL12p35 *p* = 0.139 *p* = 0.749 *p* = 0.109 *p* = 0.078

EBi3 *p* = 0.0026 *p* = 0.262 spl>sarc *p* = 0.004 spl> sk *p* = 0.004

IL2 *P =*0.0034 *p* = 0.999 spl>sarc *p* = 0.004 spl>sk *p* = 0.004

IL4 *P =*0.0021 *p* = 0.108 spl>sarc *p* = 0.004 spl>sk *p* = 0.028

IL17 *P =*0.029 *p* = 0.574 sarc>spl *p* = 0.020 sk>splp=0.020

TGF_beta *p* = 0.0011 sarc > sk *p*= 0.004 *p* = 0.025 spl>sk *p* = 0.004

IL10 *p* = 0.0017 *p* = 0.078 spl>sarc *p* = 0.004 spl>sk *p* = 0.004

FoxP3 *p* = 0.0047 sarc > sk *p*= 0.006 *p* = 0.260 spl>sk *p* = 0.007

Yellow = no overall difference between tissues

Magenta = sarcoid significantly greater than skin

Green = spleen significantly greater that sarcoid

Red = spleen significantly greater that sarcoid

Cyan = spleen significantly less than skin and sarcoid
