# Supplementary material for: Both tumour cells and infiltrating T-cells in equine sarcoids express FOXP3 associated with an immune-supressed cytokine microenvironment
Source: Vet Res. 2016 May 9;47:55. doi: 10.1186/s13567-016-0339-8 (PMC4862206; doi:10.1186/s13567-016-0339-8)
Supplement: Supplementary file 3 — 10.1186/s13567-016-0339-8 Data for validation data for FOXP3 staining with ab10563. (abCam UK). Peptide specificity of the antibody and additional control slides of various tissues are presented. [file 13567_2016_339_MOESM3_ESM.docx]

Rabbit anti Human FOXP3 (ab10563 abCam UK)

C-terminal sequence of Human FOXP3 used as immunogen.

Antibody reacts with recombinant human but not mouse FOXP3. As the antibody also reacts with samples from pigs and horses it is most likely direct at an epitope encompassing the N-terminal peptides of human FOXP3 which is predicted to cross react with Horse. The location of the conserved proprotein convertase motif is highlighted in blue and this antibody will not recognise cleaved isoforms of FOXP3.

EKGAVWTVDELEF**RKKR**SQRPSRCSNPTPGP Human

EKGAVWTVDEFEF**RKKR**SQRPSRCSNPTPGP Equine

EKGAVWTVDEFEF**RKKR**SQRPSRCSNPTPGP Pig

EKGAVWTVDEFEF**RKKR**SQRPNKCSNP--CP Mouse

Rat anti mouse FoxP3 (FJK16s eBiosciences)

This antibody recognises an epitope which maps to residues 75-125 in mouse FoxP3 which includes exon 2. Exon2 is variably spliced in human FOXP3 and spliced variants are not recognised by FJK16s. Exon 2 is not spliced in mouse so FJK16s recognises all murine Foxp3. The existence of splice variants in equines has not yet been determined.

Although FOXP3 originally came to prominence as a marker for regulatory T-cells it was soon apparent that it can be expressed in other contexts and that FOXP3 expression differed between humans and rodent [39]. Several splice variants have been demonstrated in human T-cells as well as epigenetic effects and post-translational processing which influence its function in different tissues [38, 39, 60]. Part of the ongoing controversy about FOXP3 expression may be traced to the use of antibodies that bind to epitopes affected by such changes providing apparently contradictory results. Notably the rabbit anti FOXP3 antibody (ab10563 abCam UK) did not detect any positive cells in an uninflamed equine lymph node but stained numerous cell nuclei in inflamed lymphoid tissue, the epitope recognised by this antibody lies at the extreme C-terminal of Human (and equine) FOXP3 and can be removed by post translational proteolytic cleavage at the ^414^RKKR^417^ motif. The removal of the C-terminal stabilised human FOXP3 and enhanced the regulatory function of T-cells [38]. In contrast rat monoclonal anti-mouse Foxp3 (FJK-16s s) did detect staining of lymphocytes in resting lymph nodes but not in inflamed lymphoid tissue. The epitope recognised by FJK16s has been mapped to residues 75-125 of mouse FoxP3 covering exon 2 which is differentially spliced in humans [39] (and may also be in Equines). Deletion of exon 2 has a marked effect on FOXP3 regulatory function and the role of FOXP3 in tumour development has been linked to this isoform [61, 62]. Although differential splicing of FOXP3 exon 2in the horse is supported by the findings in this paper and would providing a possible explanation for the different staining pattern obtained with these two antibodies, corroborating evidence is still required to enable any firm conclusions to be drawn.
