# Supplementary figures and images for: Both tumour cells and infiltrating T-cells in equine sarcoids express FOXP3 associated with an immune-supressed cytokine microenvironment
Source: Vet Res. 2016 May 9;47:55. doi: 10.1186/s13567-016-0339-8 (PMC4862206; doi:10.1186/s13567-016-0339-8)

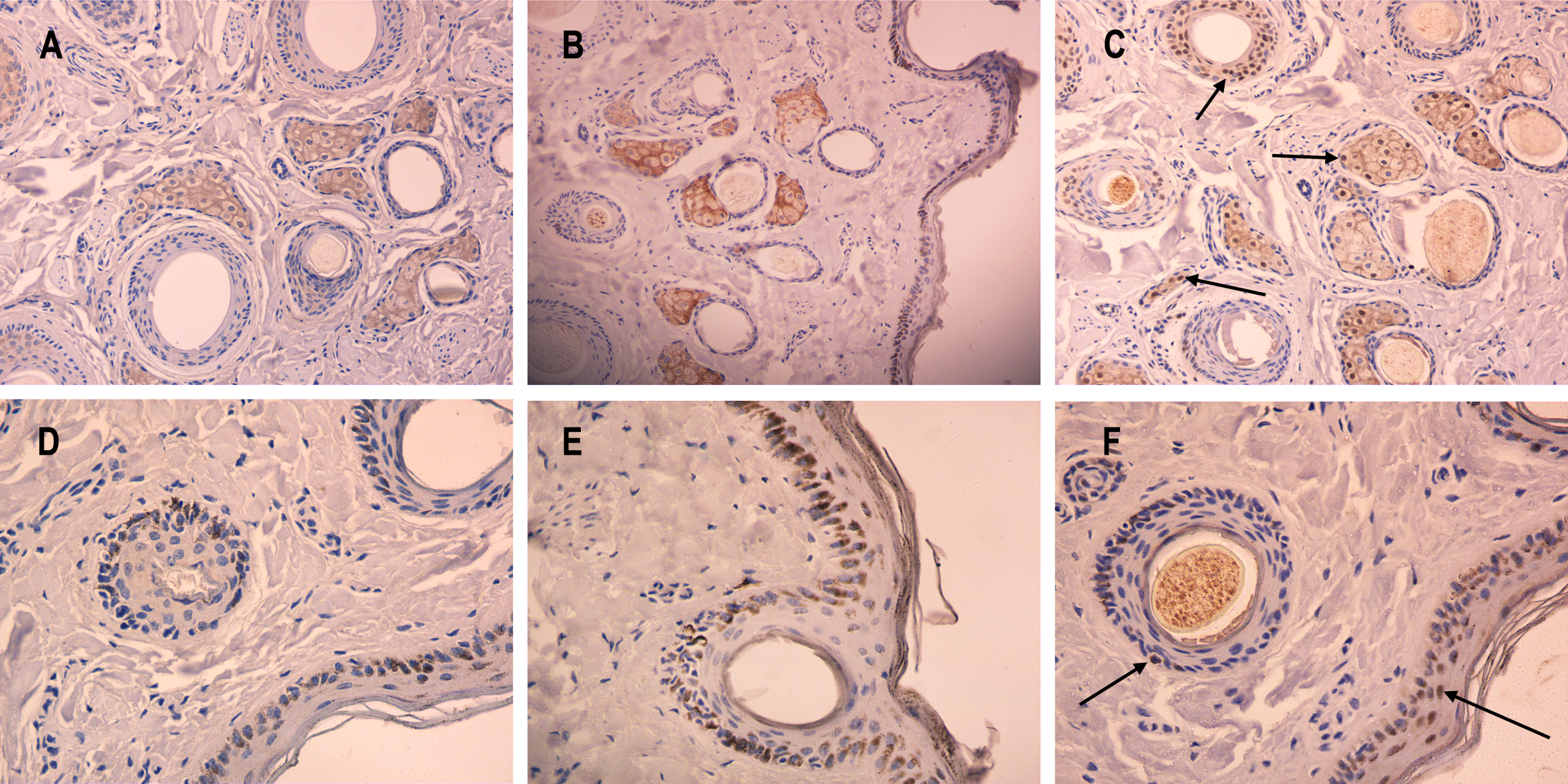

Supplement: Supplementary file 4 — 10.1186/s13567-016-0339-8 Images of FOXP3 staining in normal equine skin using (ab10563 abCam, UK), (FJK-16s eBioscience) and normal rabbit serum. Images A and D, control sections with normal rabbit serum show some background staining of cytoplasm in sebaceous glands (this type of background is typical of many antibodies). Also brown melanin granules are visible in the basal epidermis but nuclei are unstained (blue colour). Sections B and E show normal equine skin stained with Rat anti mouse FoxP3 (FJK-16s). The sections have the same background brown coloration of sebaceous gland cytoplasm as well as melanin containing hair shafts and basal cells but again there is no clear staining of nuclei. Sections C and F sow equine skin stained with Rabbit anti FOXP3 antibody (Ab 10563) once again showing the same background brown coloration of sebaceous gland cytoplasm, but in addition clear FOXP3 staining of the nuclei of sebaceous glands and nuclei of some cells in the stratum spinosum as well as the nuciei of a proportion of cells in some sections of hair follicle. [file 13567_2016_339_MOESM4_ESM.png]

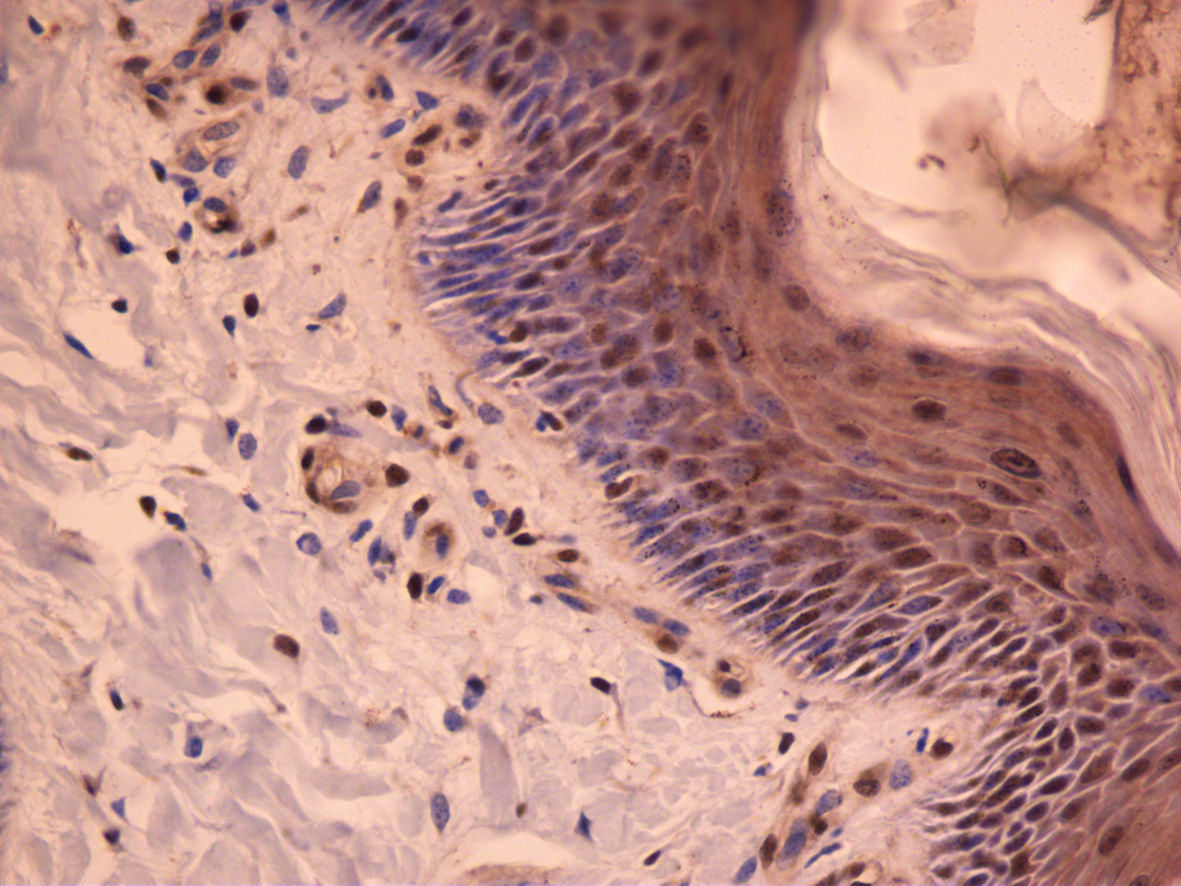

Supplement: Supplementary file 5 — 10.1186/s13567-016-0339-8 Inflamed equine skin stained with (ab10563 abCam, UK). A section of inflamed equine skin from a case of chronic chorioptic mange on the lower limb of a horse stained with Rabbit anti Human FOXP3 (ab10563 abCam UK). When stained with this antibody inflamed skin reveals more FOXP3 positive cells in the dermis and epidermis compared to normal skin. [file 13567_2016_339_MOESM5_ESM.png]

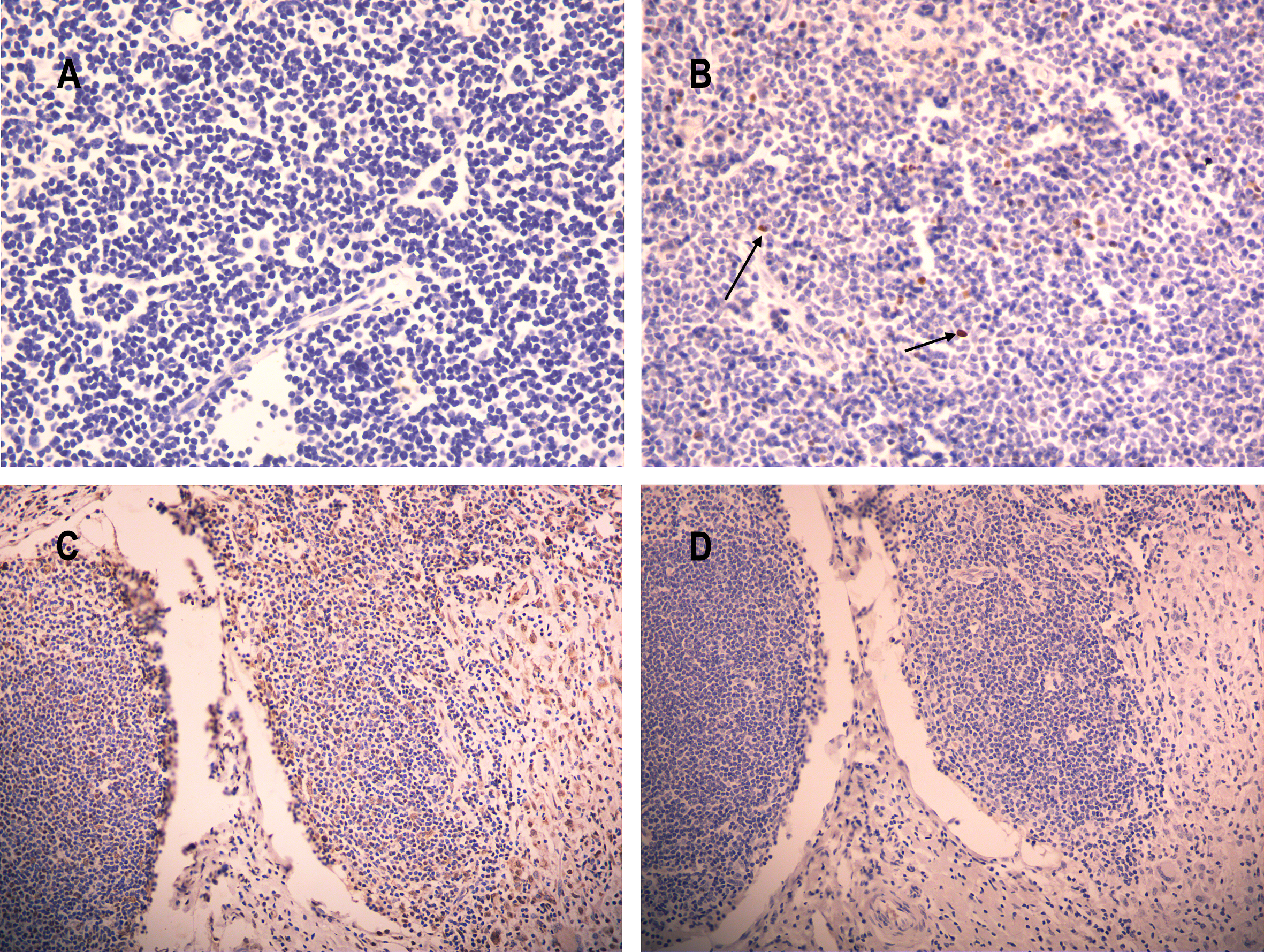

Supplement: Supplementary file 6 — 10.1186/s13567-016-0339-8 Sections of inflamed and uniflamed equine lymph node stained with (ab10563 abCam, UK) or (FJK-16s eBioscience). A The T-cell areas in an uninflamed mesenteric lymph node revealed no FOXP3 positive cells using Rabbit anti FOXP3 ( Ab ab10563 abCam UK) B The same tissue stained with rat anti-FoxP3 (FJK-16s) demonstrating scattered positive staining of lymphocytes. This contrasts with a section of inflamed lymph node in which rabbit anti FOXP3 (ab10563) stains many cells in the follicular and inter-follicular areas C, yet in this tissue FJK-16s detected no positive cells D. [file 13567_2016_339_MOESM6_ESM.png]

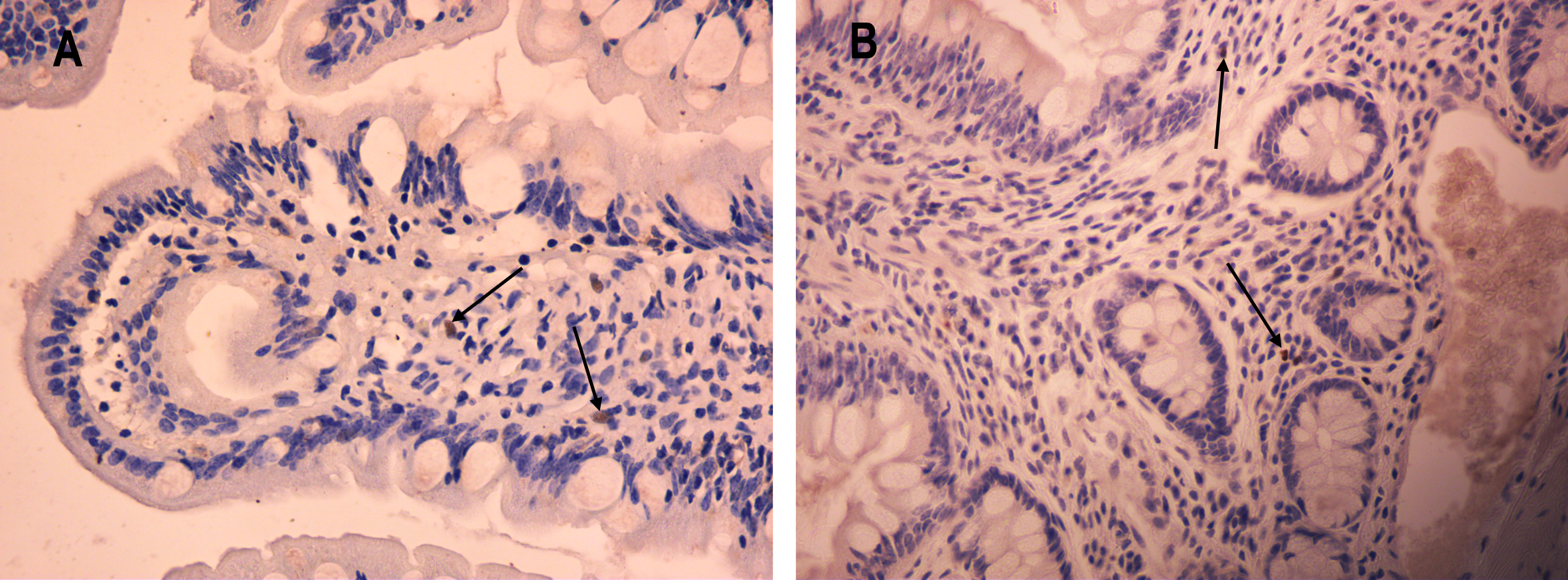

Supplement: Supplementary file 7 — 10.1186/s13567-016-0339-8 Sections of normal equine small intestine stained with (ab10563 abCam, UK) or (FJK-16s eBioscience). A rabbit anti FOXP3 and B Rat anti FoxP3 both antibodies detected nuclear staining of populations of cells in the lamina propria of equine small intestine. [file 13567_2016_339_MOESM7_ESM.png]

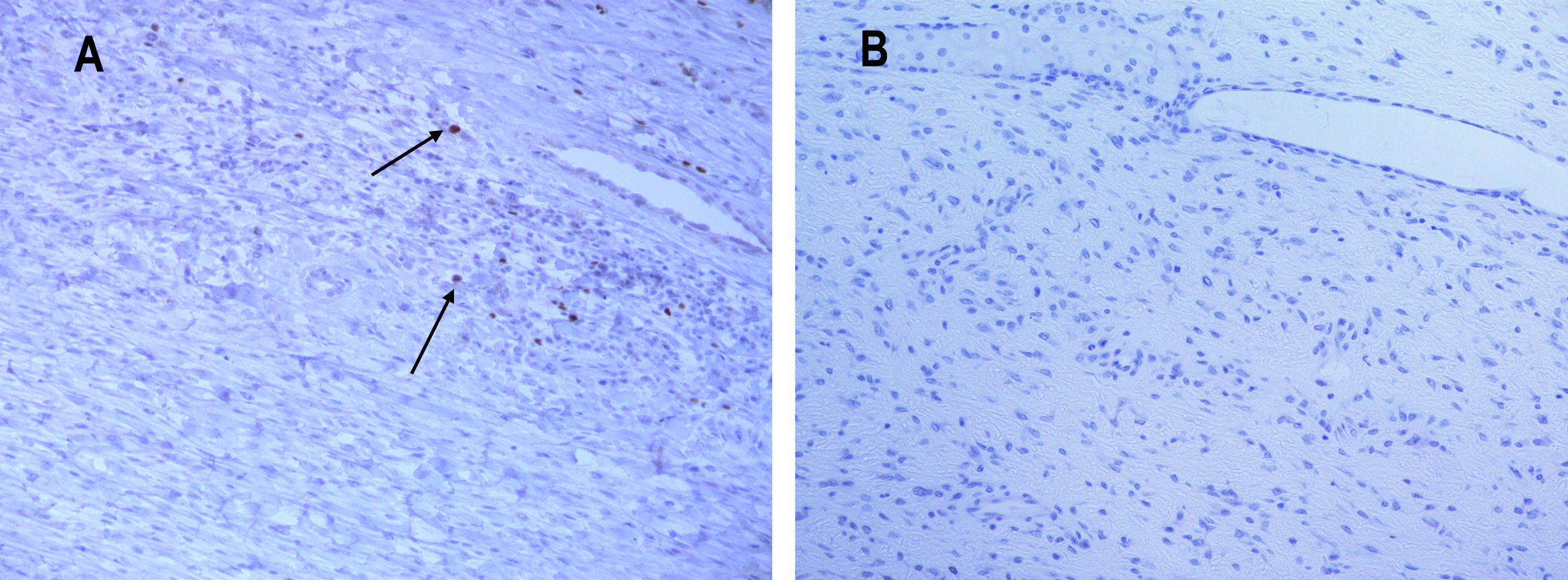

Supplement: Supplementary file 8 — 10.1186/s13567-016-0339-8 Sections of equine sarcoid stained with rat anti FoxP3. A Formalin fixed equine sarcoid stained with rat anti FoxP3 (FJK-16s eBioscience) positive stained cell were detected particularly in areas adjacent to blood vessels. B no staining was observed using isotype control antibody. [file 13567_2016_339_MOESM8_ESM.png]
