# Supplementary material for: Both tumour cells and infiltrating T-cells in equine sarcoids express FOXP3 associated with an immune-supressed cytokine microenvironment
Source: Vet Res. 2016 May 9;47:55. doi: 10.1186/s13567-016-0339-8 (PMC4862206; doi:10.1186/s13567-016-0339-8)
Supplement: Supplementary file 9 — 10.1186/s13567-016-0339-8 Table of statistical results for raw PCR data from cytokine mRNA analysis. The individual p values for all statistical comparisons between cytokine mRNA QPCR_CT number in each tissue are tabulated. [file 13567_2016_339_MOESM9_ESM.docx]

**Table of statistical analysis using raw PCR data for cytokine mRNA**

**Kruskal Wallace Mann Whitney Mann Whitney Mann Whitney**

**α = 0.05 α = 0.016 α = 0.016 α = 0.016**

**All tissues Skin vs sarcoid Sarc vs Spleen Spleen vs Skin**

IL1α *p* = 0.413 - - -

Il1β *p* = 0.010 sarc>sk *p* = 0.016 spl=sarc *p* = 0.748 spl>sk *p* = 0.004

IL6 *P* = 0.0036 sarc>sk *p* = 0.006 Spl=sarc *p* = 0.337 spl>sk *p* = 0.004

Ifnγ *p* = 0.0011 sarc>sk *p* = 0.009 spl> sarc *p* = 0.010 spl>sk *p* = 0.004

Ifnα *P* = 0.412 - - -

Ifnβ *P* = 0.269 - - -

IL12p40 *p* = 0.166 - - -

IL12p35 *p* = 0.0097 sarc>sk *p* = 0.0065 spl>sarc *p* = 0.0065 Spl>sk *p* = 0.004

EBi3 *p* = 0.0015 sarc=sk *p* = 0.057 spl>sarc *p* = 0.004 spl> sk *p* = 0.004

IL2 *P* = 0.0027 sarc=sk *p* = 0.078 spl>sarc *p* = 0.010 spl>sk *p* = 0.004

IL4 *P* = 0.0027 sar=>sk *p* = 0.0.047 spl>sarc *p* = 0.010 spl>sk *p* = 0.028

IL17 *P* = 0.042 sarc=sk *p* = 0.087 sarc>spl *p* = 0.020 sk=spl *p* = 0.370

TGFβ *p* =  0.0026 sarc>sk *p* = 0.006 spl=sarc *p* = 0.025 spl>sk *p* = 0.004

IL10 *p* =  0.0014 sarc>sk *p* = 0.006 spl=sarc *p* = 0.025 spl>sk *p* = 0.004

FoxP3 *p* =  0.0047 sarc>sk *p* = 0.006 spl=sarc *p* = 0.260 spl>sk *p* = 0.007

Yellow = no overall difference between tissues

Magenta = sarcoid significantly greater than skin

Green = spleen significantly greater that sarcoid

Red = spleen significantly greater that skin

Cyan = spleen significantly less than skin and sarcoid
